# Nanobody-mediated control of long RSH Rel and RelA catalysis by restriction of their conformational landscape

**DOI:** 10.1038/s41467-026-73059-3

**Published:** 2026-05-13

**Authors:** Katleen Van Nerom, Andres Ainelo, Kyo Coppieters ‘t Wallant, Ariel Talavera-Perez, Dannele Echemendia-Blanco, Sarah Peeters, Brahim El Khalfaoui Oulali, Hedvig Tamman, Tatsuaki Kurata, Mohammad Roghanian, Chloé Martens, Els Pardon, Jan Steyaert, Vasili Hauryliuk, Abel Garcia-Pino

**Affiliations:** 1 Cellular and Molecular Microbiology, Faculté des Sciences, Université libre de Bruxelles (ULB), Brussels, Belgium; 2 Department of Genetics, Institute of Molecular and Cell Biology, University of Tartu, Tartu, Estonia; 3 Biochemistry and Structural Biology, Université libre de Bruxelles (ULB), Bruxelles, Belgium; 4 Department of Experimental Medical Science, Lund University, Lund, Sweden; 5 RNA Systems Biochemistry Laboratory, Pioneering Research Institute, RIKEN, Wako, Saitama, Japan; 6 Department of clinical microbiology, Rigshospitalet, Copenhagen, Denmark; 7 VIB-VUB Center for Structural Biology, VIB, Pleinlaan 2, Brussels, Belgium; 8 Structural Biology Brussels, Vrije Universiteit Brussel, Pleinlaan 2, Brussels, Belgium; 9 NanoLund and Science for Life Laboratory, Lund University, Lund, Sweden; 10 University of Tartu, Institute of Technology, Tartu, Estonia; 11 WELRI: WEL Research Institute, Avenue Pasteur, 6, Wavre, Belgique

**Keywords:** Cellular microbiology, X-ray crystallography, Molecular conformation, Transferases, Ribosome

## Abstract

Long RSH enzymes, Rel and RelA, are master regulators of bacterial (p)ppGpp alarmones levels. Bifunctional Rel transitions between a compact hydrolysis-competent (HD^ON^) state, a relaxed catalytically inactive (HD^OFF^/SYNTH^OFF^) state, and an elongated synthesis-competent (SYNTH^ON^) state, whereas RelA samples only the latter two. The distribution of these states is controlled by starved ribosomes and regulatory proteins, including DarB, EIIA^Ntr^, ACP, NirD and YtfK. Here, we identify and characterize camelid nanobodies that act as selective allosteric modulators by stabilizing Rel and RelA in defined conformational states. Nanobodies that sequester the TGS domain of RelA prevent activation by deacylated tRNA on starved ribosomes, strongly inhibiting (p)ppGpp synthesis and suppressing *Escherichia coli* virulence in an animal model. Nb898 stabilizes Rel in the open SYNTH^ON^ state, enhancing synthesis while suppressing hydrolysis, whereas Nb585 traps Rel in a hydrolysis-competent HD^ON^/SYNTH^OFF^ conformation. Structural and biochemical analyses show that nanobodies, like endogenous allosteric regulators, restrict the conformational landscape of long RSH enzymes, establishing them as powerful tools for dissecting RSH function and as frameworks for developing protein-based RSH modulators.

## Abstract (web summary)

Nanobodies allosterically modulate Rel/RelA by stabilizing distinct conformations, shifting (p)ppGpp synthesis or hydrolysis. They reveal regulatory hotspots, impair stress adaptation and establish RSH enzymes as tractable targets for drug design

## Introduction

Nucleotides guanosine-3′,5′-tetraphosphate and guanosine-3′,5′-pentaphosphate—collectively referred to as (p)ppGpp alarmones—play a key role in bacterial stress sensing and adaptation^1,2^. The alarmones control different aspects of physiology, including the growth rate, metabolism, transcription and translation and are effective at different cellular concentrations^3–8^. As master regulators of bacterial physiology, the alarmones are also implicated in the control of virulence, biofilm formation and tolerance to tolerance to antimicrobials^9–13^

**Fig. 1:**
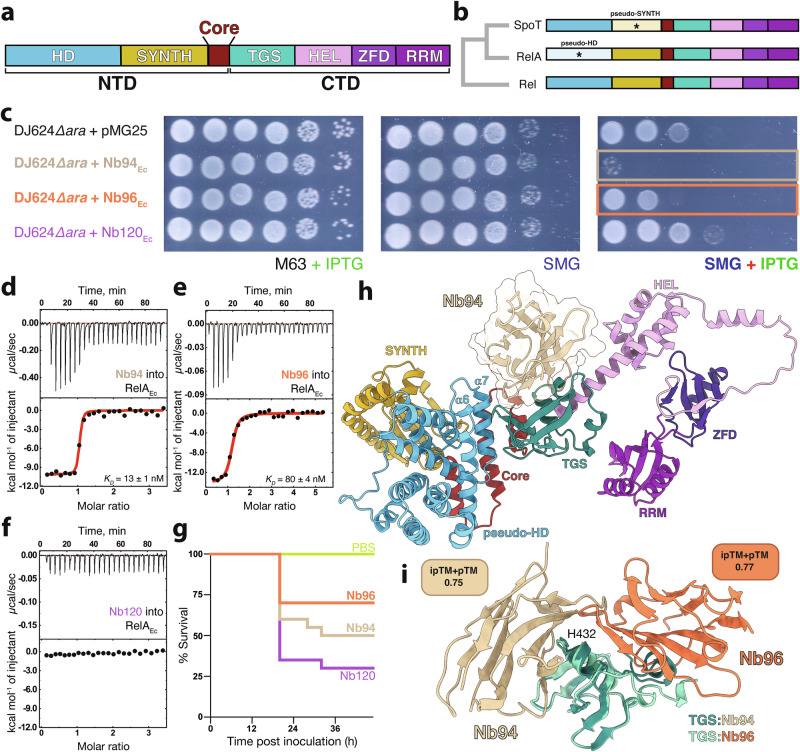
Nanobodies against RelA prevent the growth of *E. coli* under amino acid starvation conditions and curtail virulence. **a** Domain organization of long-RSH enzymes, from N to C terminus: NTDs HD (or pseudo-HD, light blue), SYNTH (in gold) and Core (in red), and CTDs, TGS (teal), HEL (pink), ZFD (dark navy blue) and RRM (purple). **b** Domain composition is conserved across long-RSH enzyme homologs. With gene duplication and specialization in monofunctional hydrolases or synthetases, one of the two catalytic domains evolved into an inactive regulatory domain (pseudo-HD or pseudo-SYNTH, represented in lighter colors). **c** SMG RelA functionality test of cells transformed with vectors expressing Nb94, Nb96 and the control Nb120 used as a negative control. ITC titrations of Nb94 (**d**), Nb96 (**e**), and Nb120 (**f**) into RelA (the insets show the average K_*D*_ ± SD for each titration, with *n* = 3). **g** Virulence assays in the *G. mellonella* infection model demonstrate that nanobodies that inhibit RelA strongly affect the virulence of *E. coli* (Source data are provided as a Source Data file). **h** AlphaFold predicted structural model of the complex of RelA (colored as in 1a) and Nb94 (in khaki). **i** Details of the interaction of Nb94 (khaki) and Nb96 (orange) with RelA’s TGS domain (colored in teal and turquoise for each respective complex). The orientation of the nanobodies in both complexes underlines the importance of H432 (labeled in the figure) to the binding interface. Prediction scores are shown in the figure. Source data are provided as a Source Data file.

The balance between synthesis and hydrolysis of (p)ppGpp is tightly controlled. The primary enzymes controlling (p)ppGpp homeostasis are long Rel/RelA-SpoT homolog enzymes, collectively known as long RSHs^14,15^. These enzymes are near-universal in bacteria, present in genomes of the majority of species, with the exception of Plantomycetes, Verrucomicrobia and Chlamydiales, the PVC superphylum^14^. Long RSHs contain two catalytic or pseudo-catalytic domains in their N-terminal half (NTD): a hydrolysis domain HD (or pseudo-HD if catalytically incompetent) and a synthesis domain SYNTH (or pseudo-SYNTH if catalytically incompetent) (Fig. 1a). Their enzymatic activity is regulated by four C-terminal regulatory domains (CTD)^16–20^ (Fig. 1a). In the case of Rel and RelA, the primary signal that stimulates (p)ppGpp production is amino acid starvation, which is sensed by recognizing the deacylated (uncharged) tRNA in the ribosomal A site^21,22^. In addition to this *in cis* intra-protein regulation, the SYNTH activity of Rel/RelA is also stimulated in trans by the alarmones themselves, with pppGpp being a more potent activator^17,23,24^.

A complex allosteric network controls and finetunes the enzymatic output of long RSH enzymes. The catalysis by the subfunctionalized RelA and SpoT RSH paralogues is evolutionarily tuned through constraints of their conformational landscape (Fig. 1b)^17–19,25^. In the case of ancestral, bifunctional Rel enzyme (capable of both degrading and synthesizing the alarmones), the opposing activities of both catalytic domains are controlled by the relative concentrations of substrates, with the ligand binding to either of the active sites switching off the activity of the other^19^. Importantly, the allosteric effect of the CTD supersedes the crosstalk between catalytic NTD domains^17,26^. In the HD^ON^ state, the enzyme is in a closed conformation, in which the CTD activates hydrolysis by stabilizing the HD domain and promoting the organization of the active site while precluding the activation of the SYNTH domain^18^. Upon amino acid starvation, binding of the enzyme to a starved (i.e., containing a deacylated tRNA in the A site) ribosome in a fully extended elongated state abolishes the allosteric effect of the CTD on hydrolysis and exposes the SYNTH domain^27,28^. However, the complete activation of the SYNTH domain also requires the binding of alarmones to a dedicated allosteric site that, acting in concert with the A-site-bound uncharged tRNA, stabilizes an elongated ribosome-bound state of the enzyme, triggering the processive synthesis of (p)ppGpp^17,26^. By contrast, active hydrolysis is contingent on a compact τ-state of long RSH enzymes Rel and SpoT, in which the SYNTH active site is occluded and the HD catalytic residues and metal cofactor are stabilized and primed for (p)ppGpp binding and hydrolysis^18,19^.

In addition to being controlled by starved ribosomal complexes, NTD and CTD regions are the target of diverse regulatory proteins that modulate the activity of RSHs^29–35^. While EIIA^NTR^, ACP, Rsd, YtfK and NirD were reported to inhibit the hydrolase function of Rel and SpoT and the synthetase activity of RelA^32,33,35–37^, DarB activates the synthetase activity of Rel in a ribosome-independent manner^38^. This suggests that long RSH enzymes possess a network of allosteric hotspots that modulate the enzymes’ catalytic activities. Such hotspots would allow them to sense the bacterial metabolic state while simultaneously providing the checkpoints that are crucial to control overproduction of (p)ppGpp^17,18^.

Camelid nanobodies are established structural biology tools used to study the conformational landscape of proteins^39–41^. Their distinct ability to recognize and stabilize specific conformational states of proteins aided by their complementarity-determining regions (CDRs)^42^ has enabled insights into dynamic and transient molecular mechanisms that are otherwise challenging to capture^43–46^. By selectively modulating allosteric sites or locking proteins in functionally relevant states, nanobodies have illuminated fundamental principles of protein folding, signaling, and catalysis across a wide range of biological systems^43–46^.

Here, we leveraged nanobodies to investigate the conformational dynamics of Rel and RelA, identifying allosteric hotspots that can be targeted to modulate their catalytic activity. Our structural and biochemical analyses reveal that by locking Rel/RelA enzymes in specific conformational states, we can precisely manipulate their activity. We have identified and characterized high-affinity nanobodies that can mimic the effect of 70S ribosomes on Rel’s synthetase activity by binding to an allosteric site on the SYNTH domain. In contrast, nanobodies that interact with the catalytic domains promote hydrolysis and strongly inhibit synthesis by stabilizing the enzyme’s τ-state. These findings highlight the potential of targeting allosteric sites outside the active centers of RSH enzymes, paving the way for antimicrobial strategies against stringent response regulators.

## Results

### Generation of high-affinity nanobodies against RSH enzymes

While the structures of the extended conformation of the ribosome-bound state of the (p)ppGpp synthetases Rel and RelA are well-characterized^27,28,47^, their off-ribosome state has remained refractory to structural biology efforts. Only the structures of the full-length monofunctional hydrolase SpoT from *Acinetobacter baumannii*^18^, which revealed a compact τ-shaped architecture incompatible with alarmone synthesis and of different truncates of Rel, have been determined in the absence of ribosomes^19,25,47–50^. To generate nanobodies for exploring the conformational landscape of long RSH synthetases off the ribosome, we immunized different llamas with *E. coli* RelA (RelA_*Ec*_) and Rel from *Chlorobaculum tepidum* (RelA_*Ct*_) and classified the resulting nanobodies according to the sequences of their third complementarity-determining region (CDR3, see Supplementary Fig. 1a).

### RelA_Ec_-targeting nanobodies inhibit (p)ppGpp synthesis by *E. coli* RelA in vivo

(p)ppGpp alarmones are essential for maintaining cellular homeostasis, specifically for controlling amino acid biosynthesis^15,51^. This key role of (p)ppGpp is leveraged in the so-called SMG functional test for RelA SYNTH activity: only *E. coli* expressing SYNTH-competent RSH can grow on SMG (minimal agar medium supplemented with Ser, Met and Gly) plates^52^. Therefore, to characterize the anti-RelA_*Ec*_ nanobodies, we have first expressed them in *E. coli* and monitored the effect on bacterial growth either under permissive conditions (M63 medium^53^, under which inhibition of RelA SYNTH activity does not affect the growth) or under non-permissive conditions (SMG medium, under these conditions inhibition of RelA SYNTH activity abrogates growth).

None of the nanobodies were toxic when expressed in bacteria growing on M63 media (Fig. 1c). Conversely, IPTG-inducible expression of Nb94 fully abrogated the *E. coli* DJ624 growth on SMG medium but not in M63 (Fig. 1c). Nb96 displayed a mild SMG-specific inhibitory effect, and Nb120, a control nanobody selected against a different target, did not inhibit growth on SMG. Next, we assessed the interactions between nanobodies and full-length *E. coli* RelA (RelA_*Ec*_) in vitro using Isothermal Titration Calorimetry (ITC). Nb94 and Nb96 bound RelA_*Ec*_ with affinities of 13 nM and 80 nM, respectively, and no interaction was detectable for Nb120 (Fig. 1d–f and Supplementary Table 1). Thus, the high affinity of Nb94 and Nb96 to RelA was consistent with the SMG assays that showed these are potent inhibitors of RelA’s SYNTH activity in vivo.

### Nb94 and Nb96 curtail the virulence of *E. coli*

(p)ppGpp-mediated signaling is crucial in antibiotic tolerance and virulence of *E. coli*. We reason that our RelA-modulating nanobodies Nb94 and Nb96 could have an impact on the virulence of *E. coli*, mimicking the effects of catalytically impaired genetic knockouts of RelA.

We used the wax moth *Galleria mellonella* larvae infection model to assess this effect on the *E. coli* clinical isolate Ec156^54^. In the presence of the control Nb120 (produced by the *Ec156_nb120* strain), which was not selected against RelA, only 30% of the larvae survived the infection after 36 h. By contrast, when the *Galleria* larvae were challenged with the *Ec156_nb94* and *Ec156_nb96* (Ec156 strains^54^ transformed with plasmids expressing the Nb94 and Nb96 nanobodies), survival increased to 50% and 70%, respectively (Fig. 1g). Notably, these strains displayed no growth defects when grown on M63 plates.

Our infection assays demonstrate that while *E.* *coli* can tolerate the loss of activity of RelA when grown in non-stressed laboratory conditions without a dramatic fitness defect; a fully functional (p)ppGpp synthetase is crucial for a successful infection.

### Nb94 and Nb96 block the TGS of RelA_Ec_

To gain a functional insight into the nanobody’s mode of action, we have used AlphaFold^55^ to model the structure of the RelA_*Ec*_:Nb94 and RelA_*Ec*_:Nb96 complexes. High confidence models predict that Nb94 and Nb96 recognize RelA_*Ec*_ via the Threonyl-tRNA Synthetase, GTPase and SpoT (TGS) domain through a ≈ 990 Å^2^ and ≈850 Å^2^ interface, respectively (Fig. 1h, i). The CDR2 and CDR3 of Nb94 and Nb96 provide the majority of the contacts (with CDR1 not involved in binding) by interfacing with the TGS domain of RelA through the central α-helical region and the C-terminal β-strand, as well as via additional contacts with the α6-α7 motif of the pseudo-HD domain and the Core domain. Interestingly, the complex interface is dominated by the H432 residue located in an antiparallel α-helical motif of the TGS (Supplementary Fig. 1a).

While in the case of Nb94, H432 makes extensive contacts with CDR2 and CDR3 and β-strands β3 and β4, in the case of Nb96, the AlphaFold-predicted mode of TGS recognition differs (Supplementary Fig. 1a, b). Nb96 engages the opposite face of the antiparallel α-helical motif within the TGS domain through all three CDRs, and additionally establishes extensive contacts via β-strands β3, β4, and β5. Notably, both nanobodies interact directly with residue H432, although in the case of Nb96, this interaction occurs specifically through CDR2. (Fig. 1i and Supplementary Fig. 1b). These differences in recognition reflect substitutions within the complementarity-determining regions, particularly in CDR2 (Supplementary Fig. 1c). Nevertheless, as both Nb94 and Nb96 nanobodies target the TGS domain of RelA, they are likely to disrupt the allosteric crosstalk between the catalytic and regulatory regions. Furthermore, their binding provides a structural basis for the potent inhibition of synthesis observed with Nb94 and Nb96, as they are predicted to tether the TGS domain to the pseudo-HD and Core domains, thereby likely preventing the enzyme from adopting its active, open conformation^27,28^, while also blocking the recognition of uncharged tRNAs on the A site by the TGS domain, which is mediated by H432^56^. We used ITC to monitor the interaction of Nb94 and Nb96 with an H432E-substituted version of RelA_*Ec*_ (RelA_*Ec*_^H432E^), a well-characterized variant that abrogates RelA’s functionality by abolishing RelA activation by deacylated tRNA^17,56^. In both cases, the introduction of a negative charge via the H432E substitution likely destabilizes the binding interface, resulting in reduced affinity. For the interaction with Nb94, H432 is buried within a hydrophobic pocket and further coordinated by D99 and D106, while in the case of Nb96, H432 lies in proximity to D55 and RelA’s D434 (Supplementary Fig. 1b, c). Indeed, the lack of binding of either nanobodies to RelA_*Ec*_^H432E^ (Supplementary Fig. 1d, e) supports the proposed mode of recognition and inhibition of ppGpp synthesis through sequestration of the TGS domain.

### Nanobodies modulate the catalytic activities of Rel_Ct_

While the monofunctional SYNTH-only RelA can only explore the resting and extended conformational states, bifunctional—that is, capable of both hydrolysis and synthesis of (p)ppGpp—Rel RSH enzymes can explore a much broader conformational landscape. Rel enzymes can assume a compact HD^ON^-compatible τ-state in which both catalytic domains are close together with the synthetase active site completely occluded^18^. Furthermore, Rel can adopt a relaxed conformation, which is less structured compared with the τ-state. In this state, both catalytic domains are partially open and marginally active^18,19^. Finally, Rel can also assume an elongated ribosome-bound SYNTH^ON^-compatible state, in which the NTD is fully open, and the hydrolase active site is disordered and inactive^18,19^. Therefore, identifying and engineering specific conformational modulators of Rel is likely more challenging than for the less conformationally dynamic RelA. We turned to the bifunctional (p)ppGpp synthetase/hydrolase Rel from the model thermophile *C. tepidum* (Rel_*Ct*_) to test whether nanobodies could selectively restrict the allosteric landscape of these flexible long RSH factors to trap the enzyme in a specific state that would define its catalytic output.

**Fig. 2:**
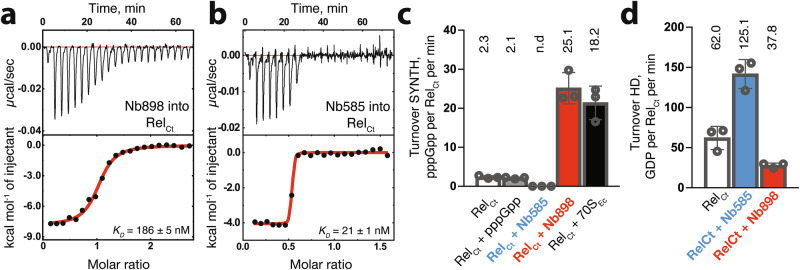
Nanobodies targeting Rel can modulate the activity of the enzyme by restricting their allosteric landscape. ITC titrations of Nb898 (**a**), Nb585 (**b**), into Rel_*Ct*_ (the insets show the average K_*D*_ ± SD for each titration, with *n* = 3). **c** SYNTH activity of Rel_*Ct*_ unbound (empty bar); in the presence of Nb898 (red bars); Nb585 (blue bars); pppGpp (gray) and 70S ribosomes (black). **d** HD activity of Rel_*Ct*_ unbound, in the presence of Nb898 (red bars), Nb585 (blue bars). The contrasting features of the action of the nanobodies in (**c**) and (**d**) suggest they hijack the enzyme’s conformational landscape to favor one activity at the detriment of the other. Data are presented as mean values ± SD, shown as error bars (with *n* = 3). Source data are provided as a Source Data file.

Using the same strategy to identify nanobodies targeting RelA_*Ec*_, we selected two different families of nanobodies—Nb898 and Nb585—that strongly bind Rel_*Ct*_ (Supplementary Fig. 2). ITC showed that full-length Rel_*Ct*_ interacts with Nb898 and Nb585 with *K*_D_ values of 186 nM and 21 nM, respectively (Fig. 2a, b and Supplementary Table 1). In both cases, this interaction is confined to the N-terminal catalytic region (NTD) of the enzyme since C-terminal truncations did not affect binding (Supplementary Table 1). In the absence of Rel SYNTH stimulators, pppGpp or 70S *E. coli* ribosomes, Rel_*Ct*_ is a poor catalyst of ppGpp synthesis (Fig. 2c). Addition of *E. coli* 70S ribosomes and pppGpp increases the SYNTH activity ≈8 times. In the presence of Nb898, the turnover of Rel_*Ct*_ increased ≈11-fold, on par with the enhancing effects of the 70S and pppGpp combined. This effect is accompanied by a 1.6-fold decrease in hydrolase activity (Fig. 2d) that results in the accumulation of ppGpp. By contrast, Nb585 turns the SYNTH domain off, strongly inhibiting (p)ppGpp synthesis (Fig. 2c) while increasing the hydrolase activity of Rel_*Ct*_ by two-fold (Fig. 2d). These results are consistent with the nanobodies modulating the antagonistic allosteric coupling between the two catalytic domains of Rel_*Ct*_ to control the enzymatic output.

### Nb898 recognizes Rel_Ct_^NTD^ SYNTH domain

We monitored the structural effects of Nb898 binding using Hydrogen Deuterium eXchange coupled with Mass Spectrometry (HDX-MS)^57^ by comparing Rel_*Ct*_^NTD^ (a more stable and soluble fragment that contains the Nb binding site) in its substrate-free unliganded state, and in the Rel_*Ct*_^NTD^:Nb898 complex (Supplementary Fig. 3a-i and Supplementary Table 2).

The overall effect of Nb898 binding was a prominent decrease in deuterium exchange in the region that contains α-helix α13 (residues E279 to Y290), which is indicative of the direct association of Nb898 with Rel_*Ct*_^NTD^ and showed additional deuterium protection signal in the hydrolase active site (residues 65-77, Supplementary Fig. 3b, g-i). This region involves part of the HD active site of Rel_*Ct*_, suggesting a possible allosteric stiffening of this active site in the SYNTH^ON^ state, as observed in other Rel enzymes^19,48^.

**Fig. 3:**
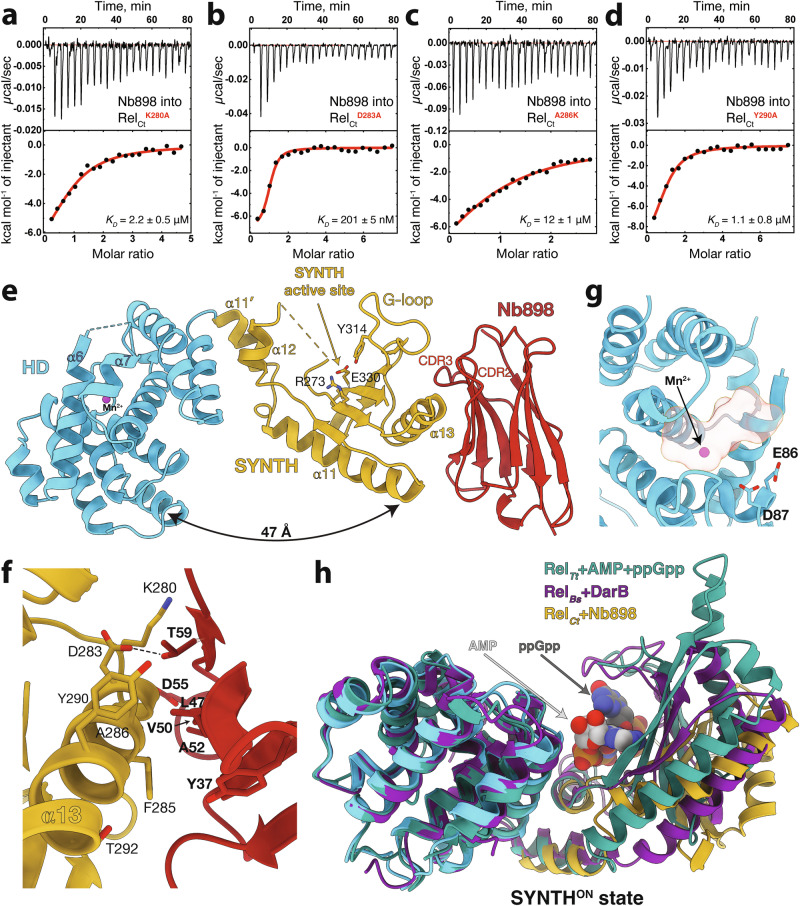
Nb898 binds to the SYNTH domain of Rel_*Ct*_ contacting α13 and the G-loop. ITC titrations of Nb898 into Rel_*Ct*_^K280A^ (**a**), Rel_*Ct*_^D283A^ (**b**), Rel_*Ct*_^A286K^ (**c**), and Rel_*Ct*_^Y290A^ (**d**) (the insets show the average K_*D*_ ± SD for each titration, with *n* = 3). into Rel_*Ct*_ to validate the binding interface. **e** Crystal structure of the Rel_*Ct*_^NTD^-Nb898 complex. Rel’s HD and SYNTH domains are colored as in Fig. 1a, and Nb898 in red. CDR2 and CDR3 are labeled. **f** Details of Rel_*Ct*_^NTD^-Nb898 binding interface highlighting key structural elements. **g** Details of Rel_*Ct*_ HD- domain highlighting as a surface the (p)ppGpp binding site and displaying the position of the Mn^2+^ ion and the catalytic residues E86, D87 in the active site. **h** Superposition of the catalytic domains of Rel_*Ct*_ onto the Rel_*Tt*_-AMP-ppGpp complex (PDBID 6S2U, in green) and the Rel_*Bs*_-DarB complex (PDBID 8ACU, in purple). The superposition confirms Nb898 selects the open, SYNTH^ON^ conformation of the enzyme. The AMP and ppGpp nucleotides observed in the post-catalytic state of Rel_*Tt*_ are highlighted with light and dark gray arrows, respectively. Source data are provided as a Source Data file.

To validate the HDX-identified binding interface, we targeted the epitope defined by α13 for point substitutions (K280A, D283A, F285A, A286K, Y290A), and monitored the interaction of Rel_*Ct*_ variants with Nb898 by ITC. With the exception of D283A, which resulted in only a minor change in the *K*_D_, in all cases we detected a decrease in affinity as compared to the WT Rel_*Ct*_ (Fig. 3a-d and Supplementary Table 1), thus validating the binding epitope. Collectively, these results indicated that the direct interaction of Nb898 with the SYNTH domain triggered the closing or rigidification of HD and consequent inactivation of the hydrolase function and allosterically activated the SYNTH function.

### Nb898 stabilizes the SYNTH^ON^ active state of Rel_Ct_^NTD^

In order to understand the effect of Nb898 on catalysis, we determined the structure of the Nb898:Rel_*Ct*_^NTD^ complex (Fig. 3e, Supplementary Fig. 4a–d, and Supplementary Table 3). The structure confirms the binding interface predicted by the HDX-MS: indeed, Nb898 engages Rel_*Ct*_^NTD^ via the SYNTH domain. The interactions from the nanobody side occur mainly through the core β-sheet of the Ig-domain, CDR2, which forms a flat hairpin structure with a pronounced bulge in the middle, stacking α13, plus additional contribution from CDR3 (Fig. 3e). This binding interface is in perfect agreement with HDX and ITC (Supplementary Fig. 3 and 3a–d). The stacking interface is largely hydrophobic, composed of the side chains of residues I281, F285, A286, Y290 and T292 from the Rel_*Ct*_ side and Y37, A47, V50, A52, D55 and T59 from Nb898. In addition, T59 forms a hydrogen bond with D283, tethering CDR2 to α13 and the backbone of T58, and forms a hydrogen bond with K280 (Fig. 3f).

In the conformation of Rel_*Ct*_^NTD^ induced by Nb898, the HD and SYNTH domains are distanced 47 Å (Fig. 3e). The active site of the HD domain is largely misaligned with the ED catalytic motif away from the Mn^2+^ ion and the pocket that coordinates the 3′-pyrophosphate of (p)ppGpp during hydrolysis (Fig. 3g). By contrast, the SYNTH domain active site and catalytic residues are exposed to the solvent. This is accompanied with the fracturing of α11 (into α11′ and α11) and the displacement of α12, all signature structural elements of the activation of catalysis by the SYNTH domain^19^ (Fig. 3e). Noteworthy, this mode of recognition via α13 mimics that of *Thermus thermophilus* Rel (Rel_*Tt*_) bound to its SYNTH reaction products ppGpp and AMP^19^ (Fig. 3h) as well as of *B. subtilis* Rel (Rel_*Bs*_) bound to DarB in absence of cAMP^48^ (Fig. 3h and Supplementary Fig. 4e-f); the latter also mediates the activation of ppGpp synthesis and inhibition of hydrolysis^48^ (Fig. 3h).

**Fig. 4:**
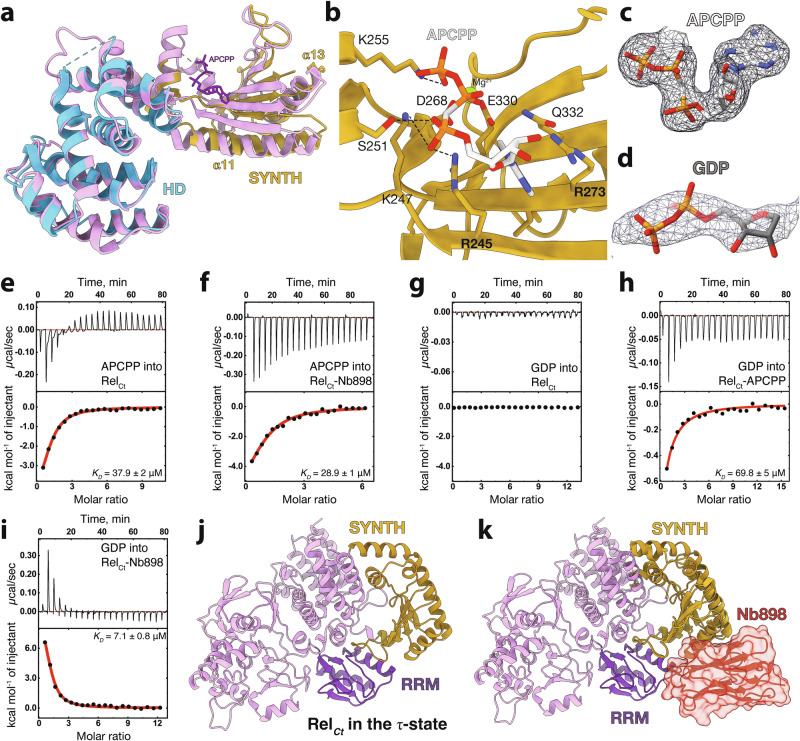
Nb898 binds to the SYNTH domain of Rel_*Ct*_ and allosterically enhances the affinity of the enzyme for GDP. **a** Superposition of the structure of the Rel_*Ct*_^NTD^-Nb898 complex (colored as in Fig. 1a) on the structure of Rel_*Ct*_^NTD^ (in pink) in complex with APCPP (in purple). Crucial structural elements involved in the ON/OFF switch of the enzyme, such as α11, α13 are labeled in the figure. The superposition confirms Nb898 triggers an active state of Rel_*Ct*_^NTD^ compatible with nucleotide binding. **b** Details of Rel_*Ct*_^NTD^-APCPP complex interface highlighting key active site residues involved in substrate binding and catalysis. Simulated annealing composite omit electron density maps at 1.0σ corresponding to the observed ligands bound to Rel_*Ct*_^NTD^, APCPP (**c**), ribose 5′-pyrophosphate group of GDP (**d**) as observed in chain A. ITC titration of APCPP into Rel_*Ct*_^NTD^ (**e**); APCPP into Rel_*Ct*_^NTD^-Nb898 complex (**f**); GDP into Rel_*Ct*_^NTD^ (**g**); GDP into Rel_*Ct*_^NTD^-APCPP complex (**h**); GDP into Rel_*Ct*_^NTD^-Nb898 complex (**i**). The insets for each titration show the average K_*D*_ ± SD (with *n* = 3). **j** Cartoon representation of a model of full-length Rel_*Ct*_ in the hydrolase-compatible τ state. The SYNTH (in gold) and RRM (purple) domains are labeled in the figure. **k** Surface representation of an idealized (unrealistic) model of full-length Rel_*Ct*_^NTD^ in complex with Nb898 (in red). The model confirms that the Rel_*Ct*_^NTD^-Nb898 complex is incompatible with the hydrolase-active τ state. Source data are provided as a Source Data file.

Catalysis by long RSH enzymes is directly linked to their conformational state^18,28^. Binding of GDP and ATP triggers the opening of the NTD region, exposing the SYNTH active site, while the binding of ppGpp exposes the HD domain and inactivates SYNTH^19^. Using GDP and a nonhydrolyzable ATP analog adenosine-5′-[(α,β)-methyleno]triphosphate, APCPP, to induce the active SYNTH state of Rel_*Ct*_^NTD^, we determined the structure of the substrate-liganded complex at 2.65 Å. The binding of both nucleotides, indeed, promotes the open conformation (Fig. 4a and Supplementary Table 3), with APCPP buried in the active site and GDP more exposed (Fig. 4b and Supplementary Fig. 4g). The adenine base of APCPP is coordinated via π-π interactions by R245 and R273, whereas the triphosphate moiety is held in position by interactions with R245, K247, S251 and K255 (Fig. 4b). At the same time, GDP is stabilized by residues K255, K261, and K303. However, while the APCPP density is well-defined (Fig. 4c), GDP is only partially resolved: strong electron density is observed only for the ribose 5′-pyrophosphate in chain A (Fig. 4d) and the pyrophosphate group in the others (Supplementary Fig. 4g–i). The APCPP- and GDP-bound open state of Rel_*Ct*_^NTD^ matches well the conformation of Rel_*Ct*_^NTD^ observed in the complex with Nb898 (Fig. 4a), supporting the idea that Nb898 stabilized the SYNTH^ON^ conformation of Rel_*Ct*_^NTD^ even in the absence of substrates.

### Nb898 stimulates SYNTH activity Rel_Ct_^NTD^ by promoting nucleotide binding

RSH enzymes typically display a preferential order for the incorporation of substrates to the SYNTH active site^19,48,58^. We used ITC to probe the incorporation of nucleotides into Rel_*Ct*_^NTD^. Rel_*Ct*_^NTD^ binds the APCPP with an affinity of 37.9 μM, which is enhanced by 1.3-fold in the presence of Nb898 (Fig. 4e, f). By contrast, in the absence of APCPP or Nb898, we were not able to detect binding of GDP to Rel_*Ct*_^NTD^ (Fig. 4g). In the presence of saturating APCPP concentration (5-fold above the *K*_D_), the enzyme binds the GDP substrate with a *K*_D_ of 69.8 μM, and Nb898 has a 10-fold stimulatory effect on the interaction (*K*_D_ = 7.0 μM) (Fig. 4h, i).

Interestingly, the binding of GDP to Rel in the presence of APCPP is enthalpically driven, whereas Nb898 induces a clear switch to an entropically driven interaction (Supplementary Table 1). This reflects the allosteric exposure of hydrophobic patches within the large SYNTH active site (including the G-loop), which are associated with highly ordered water molecules. Upon GDP binding to the Rel_*Ct*_-Nb898 complex, release of these waters into bulk solvent likely generates a substantial entropic gain that dominates the binding free energy. Moreover, Nb898 likely pre-organizes Rel into a more restricted, bound-like conformational ensemble, thereby minimizing the conformational entropy penalty associated with complex formation. Such changes in binding energetics are consistent with enthalpy-entropy compensation commonly observed upon perturbation of biomolecular recognition.

Collectively, these results indicate that Rel_*Ct*_^NTD^ binds its substrates in a sequential and ordered manner, initiated by ATP and followed by GDP or GTP. In addition, they suggest that the stabilizing presence of Nb898 enhances the affinity of the enzyme for both nucleotides, which underlines the SYNTH-stimulatory effect of the nanobody.

### Nb898 modulates Rel_Ct_ τ-state precluding CTD autoinhibition and HD activation

To further investigate the mechanism of allosteric activation of Rel_*Ct*_ by Nb898, we modeled the structure of unliganded full-length Rel_*Ct*_ based on the structure of the highly compact τ-state of *A. baumannii* SpoT (SpoT_*Ab*_) which represents a CTD-autoinhibited long RSH primed for hydrolysis (Fig. 4j). Indeed, the integrity of the association of the CTD of long RSHs with its HD and SYNTH domains is crucial for the stability of the SYNTH-inhibited τ-state (SYNTH^OFF^) of SpoT and Rel as evidenced by a decrease in their hydrolase activity upon destabilizing mutations or domain deletions^18,26,59^. By contrast, the regulation of SYNTH activity follows a distinct mechanism. Activation of Rel and the role of the CTD in restraining this activity are only physiologically relevant in the context of starved ribosomes. In this setting, CTD-mediated autoinhibition represents just one component of a multistep process that also requires activation and stabilization of the fully active enzyme through interactions with A-site deacylated tRNAs and pppGpp.

While the CTD itself is intricately involved in ribosome binding, in the absence of ribosomes, CTD truncations do exhibit an increase in (p)ppGpp synthesis^20,59,60^. In addition, as observed for the Rel activator DarB^48^, a ribosome-independent stabilization of an open, elongated state of Rel could have an impact on the SYNTH activity of the enzyme^48^. When the structure of the Rel_*Ct*_-Nb898 complex is compared with the model of SYNTH^OFF^ Rel_*Ct*_ it becomes apparent that the two states are incompatible. The strong binding of Nb898 would directly interfere with the RRM:SYNTH association, which would drive the conformational equilibrium of the enzyme toward a relaxed non-autoinhibited state more compatible with SYNTH activity (Fig. 4k). Collectively, these results suggest that Nb898 hijacks a naturally existing allosteric pathway of activation of long RSH factors. In this way, Nb898 restricts the conformational landscape of the enzyme to the SYNTH open state, allosterically priming catalytic residues and enhancing substrate recognition, which, in turn, stimulates (p)ppGpp synthesis.

### Nb585 locks Rel_Ct_ and primes the HD domain while switching off the SYNTH domain

**Fig. 5:**
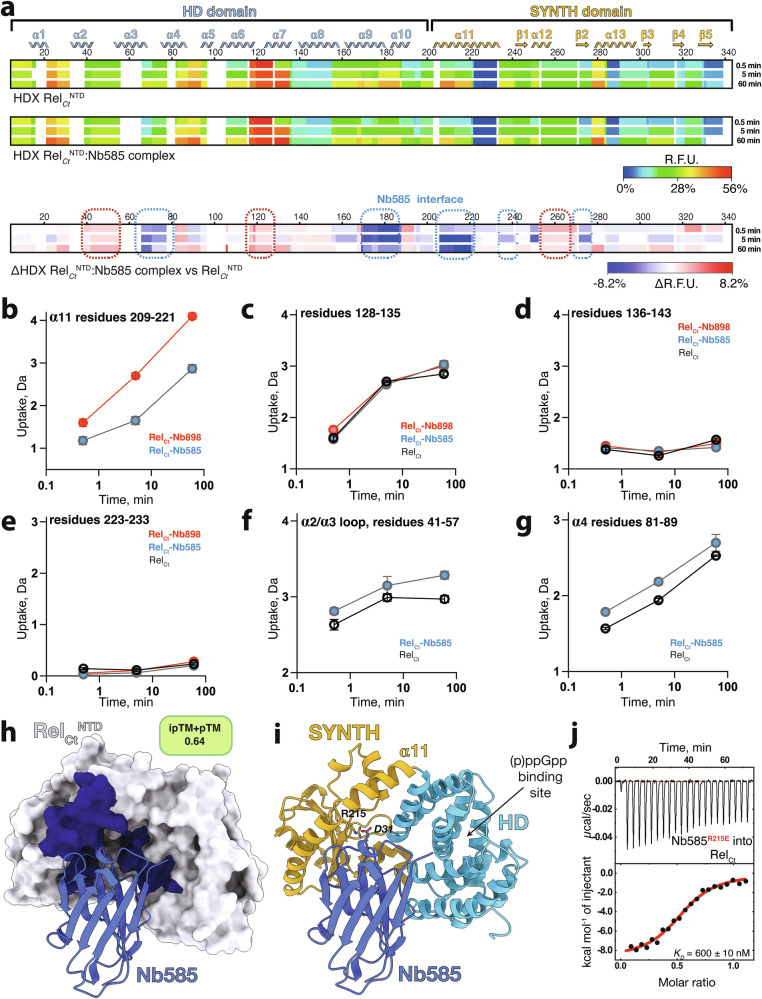
Nb585 contacts both HD and SYNTH domains of Rel_*Ct*_ stabilizing a closed conformation that is compatible with ppGpp hydrolysis. **a** Heatmaps representing the HDX of Rel_*Ct*_^NTD^ (top), Rel_*Ct*_^NTD^-Nb585 complex (center) and ΔHDX (bottom). Residues involved in the binding interface are outlined by a dashed light blue line, and regions displaying high uptake are outlined by a dashed dark red line. **b** Deuterium uptake profile for a representative peptide, the allosteric hotspot region involving residues 209–221 of α11 of Rel_*Ct*_^NTD^-Nb585 complex in blue vs Rel_*Ct*_^NTD^-Nb898 in red. Deuterium uptake profile of control peptides 128–135 (**c**); 136–143 (**d**), and 223–233 (**e**), representative of regions with negligible exchange. Deuterium uptake profile for a representative peptide of the α2/α3 loop (**f**) and the active site fragment of α4 (**g**). In all cases, uptake profiles are presented as mean values ± SD, shown as error bars (with *n* = 3). **h** Surface representation of Rel_*Ct*_^NTD^ (in light gray) bound to Nb585 (in blue) as predicted by AlphaFold. Rel_*Ct*_^NTD^ residues involved in the binding interface are colored in dark blue. **i** Cartoon representation of the model of Rel_*Ct*_^NTD^-Nb585 complex with Rel_*Ct*_^NTD^ HD and SYNTH domains colored as in Fig. 1a and Nb585 in blue. The Mn^2+^ ion, a proxy for the (p)ppGpp binding site in the HD domain, is highlighted in the figure. **j** ITC titration of Nb585 R215E substituted version (Nb585^R215E^) into Rel_*Ct*_^NTD^ (the inset shows the average K_*D*_ ± SD, with *n* = 3). This position is predicted by AlphaFold and HDX-MS to be involved in the complex interface. The increase in K_*D*_ triggered by the R215E substitution supports the predicted mode of action of Nb585. RFU, relative fractional uptake. Source data are provided as a Source Data file.

As with the SYNTH-activating Nb898, we used HDX-MS to characterize the interaction of the HD-activating Nb585 with Rel_*Ct*_. In the presence of Nb585, the central region of the enzyme that comprises the C-terminal part of the HD domain that mediates the coupling between SYNTH and HD domains and α11, displayed a strong deuterium protection signal (Fig. 5a, b); differing from other regions that showed no exchange (Fig. 5c–e). These structural elements are all solvent-exposed in the open pre-catalytic state of Rel_*Ct*_ when bound to GDP and APCPP and in the complex with Nb898. However, these regions coalesce together around the central axis of the enzyme in a closed state and become protected from deuterium exchange (Fig. 5a, b). By contrast, in the HD domain the active site shows a moderate increase in deuterium uptake consistent particularly in the regions containing K47-R48 involved in guanosine coordination, and H57, H82, D83 involved in Mn^2+^ coordination (Figs. 5a and 5f-g). Collectively, these results are consistent with the exposure of catalytic residues upon binding to Nb585 and the stabilization of a HD^ON^ state.

The HDX-MS data are in good agreement with the AlphaFold prediction of the structure of the complex that places Nb585 between the HD and SYNTH domains. In the predicted complex, Nb585 interacts with the SYNTH domain via the region involved in ATP binding, reminiscent of the way toxic Small Alarmone Synthetases (toxSAS) are inhibited by their antitoxins^61^, which is consistent with the strong inhibition of synthesis displayed by Nb585. This binding mode tightly tethers the HD and SYNTH domains (Fig. 5h–j), stabilizing a closed state that resembles that of Rel_*Tt*_ bound to ppGpp^19^ (*r.m.s.d* of 0.9 Å, Supplementary Fig. 5a). According to the model and consistent with the HDX-MS data, Nb585 makes extensive contacts with the C-terminal α-helix of the HD domain and α-helix α11 of SYNTH. In particular, R215 of Rel_*Ct*_ is predicted to form a salt bridge with D31 of Nb585 (Supplementary Fig. 5b, c). This region contains the kink implicated in the closed/open switch of the NTD and is strongly protected from deuterium exchange upon Nb585 binding (Fig. 5a-b). To test this, we introduced the R215E substitution in Rel_*Ct*_ and observed a 28-fold drop in affinity of Nb585 for Rel_*Ct*_^R215E^ (Fig. 5j). This is consistent with our model of the complex (Fig. 5h) and the HDX-MS and biochemical data (Figs. 2c, d and 5a). Collectively, these results suggest that long RSH-binding molecules that activate one catalytic function likely trap the enzyme in a restricted conformational state that precludes the opposing catalysis.

## Discussion

As the stringent response is a key regulatory pathway implicated in virulence and antibiotic resistance, chemical inhibition of RSH enzymes presents an attractive strategy for the development of antibacterials that disrupt the (p)ppGpp homeostasis^62^. Here, we identified nanobodies that are efficient allosteric modulators of long SYNTH-competent ribosome-associated long RSH Rel and RelA and dissected their mechanism of action. We showed that these nanobodies are effective tools to control the catalytic output of these enzymes in vitro and in vivo, precluding bacterial growth under nutrient starvation.

**Fig. 6:**
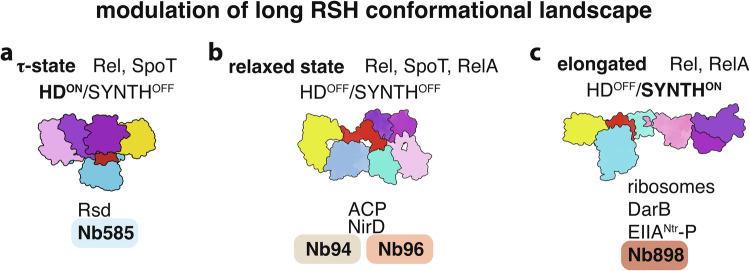
Naturally occurring allosteric sites of the RSH enzyme can be exploited to modulate their activity. Cartoon representation of observed conformational states of long-RSH enzymes (τ, relaxed and elongated)^18^. These different states are stabilized by cellular binding partners to modulate the RSH-output (the different enzymatic domains are colored according to Fig. 1a). Nanobodies such as Nb585 (**a**), Nb94 (**b**), and Nb898 (**c**) bind to Rel and RelA mimicking the mode of action of endogenous RSH adaptor proteins that are activated during the cell cycles to modulate the metabolic state. The action of these nanobodies suggests that, besides the well-characterized genetic effects on antibiotic tolerance, virulence and survival, chemically targeting RSH enzymes could be a viable pathway to develop drugs active against bacteria.

In addition to the canonical ribosome-dependent pathway, the enzymatic activities of long RSH enzymes are controlled by diverse adaptor proteins (Fig. 6a–c). In *E. coli*, Rsd is a stimulator of the (p)ppGpp-hydrolase activity of SpoT during carbon source starvation^33^ (Fig. 6a). In the absence of c-di-AMP, DarB binds strongly to the SYNTH domain of *B. subtilis* Rel^48^, while analogously, upon phosphorylation, *C. crescentus* EIIA^Ntr^ binds the RRM domain of Rel, which leads to a strong inhibition of hydrolysis^32^ and ppGpp accumulation during nitrogen starvation^31^. Both regulators preclude the formation of the τ-state of Rel^18^ and inhibit the hydrolase function by restricting the conformational space to an open, SYNTH^ON^ conformation only compatible with (p)ppGpp synthesis^32,48^. The combined allosteric effect of favoring synthesis while inhibiting hydrolysis collectively leads to the accumulation of (p)ppGpp. Finally, exploring an alternative regulatory strategy, NirD binds to both domains of RelA^NTD^ and prevents (p)ppGpp synthesis^37^ (Fig. 6b).

The nanobodies characterized in this study target key regulatory hotspots in long RSH enzymes, acting as catalytic checkpoints. Nb94 and Nb96 recognize and occlude the antiparallel α-helical motif of the *E. coli* RelA TGS domain, the element that inspects the CCA end of tRNAs and is essential for RelA’s activation on the starved ribosomal complex^27,28^. Thus, selective inactivation of the TGS domain with Nb94 and Nb96 counters the intramolecular allosteric signaling that triggers RelA-mediated (p)ppGpp synthesis in response to amino acid starvation. Analogously, Nb898 binds the structural epitope that we have earlier shown in the case of *B. subtilis* Rel to be recognized by SYNTH-stimulatory factor DarB^48^. Neither Nb898 nor DarB makes direct contact with the active site of the enzyme to enhance (p)ppGpp synthesis. Rather, both modulators counter the formation of the hydrolase-compatible τ-state by preventing the interaction between the RRM domain and the SYNTH domain (Supplementary Fig. 4e, f). This, in turn, leads to a partial opening of SYNTH (Fig. 4h-i). Indeed, binding of Nb898 enhances the affinity of the enzyme for ATP, mimicking the stimulatory effect of the 70S ribosome and resembling the genetic truncation of the RRM domain of Rel and RelA that induces slow growth by the toxic accumulation of (p)ppGpp^20^ (Fig. 6c). The stronger stimulatory effect of Nb898 as compared to DarB is likely the result of the higher affinity for Rel (186 nM in the case of Nb898 *vs* 1.4 μM in the case of DarB^48^), suggesting that tight-binding molecules targeting this particular region of Rel are likely to induce severe growth arrest. Nb585 recognizes a more complex tridimensional epitope that includes both N-terminal catalytic domains, which is reminiscent of the conformational recognition strategy employed by NirD^37^ to inhibit RelA. However, from a functional perspective, Nb585 mimics Rsd^33^ by stabilizing the closed τ-state of the long RSH enzyme to increase the hydrolase activity while inhibiting the (p)ppGpp synthesis.

Functional effects mediated by nanobodies mirror the activity-defining rearrangements stabilized by nanobodies on the cystic fibrosis transmembrane conductance regulator (CFTR)^40,46^ and GPCRs^45^. Nanobodies targeting extracellular epitopes of proteins are currently being developed as potential drugs for a variety of human diseases, including antimicrobials; however, they target mainly secreted toxins or toxin receptors^63–65^. It is important to emphasize that the nanobodies developed in this study are not intended as therapeutic agents, but rather as exploratory tools. While these nanobodies robustly modulate long RSH activity in vitro and in the *Galleria* infection model, their value lies in establishing long RSH enzymes as *bonafide* targets to develop antibacterials. By revealing discrete allosteric hotspots and defining conformational states that correlate with catalytic output, these nanobodies offer a structural and conceptual framework for the rational design and development of small-molecule modulators targeting the stringent response, and inform future studies aimed at exploiting the enzymatic output of long RSHs.

## Methods

### Ethical statement

To generate camelid antibodies, llamas were immunized, and blood samples were collected in full compliance with European legislation (EU Directive 2010/63/EU) and the Belgian Royal Decree of 29 May 2013 concerning the protection of laboratory animals. The animals were housed at a licensed facility (accreditation number LA 1700601) recognized by the Belgian competent authorities. Animal welfare was continuously monitored by an accredited veterinarian. All procedures, including immunization and blood collection, were reviewed and approved by the local Animal Ethics Committee.

### Multiple sequence alignment

Sequences were aligned with MAFFT v7.164b with the L-INS-i strategy^66^ and visualized with Jalview^67^.

### Generation of RSH-targeting nanobodies

Following established protocols^68^, peripheral blood lymphocytes were collected from the immunized llamas, and RNA was extracted for cDNA synthesis. After the first amplification with primers call001 and call002 (Supplementary Table 4), the variable domains of heavy-chain-only antibodies were amplified using primers 229 and 230 (Supplementary Table 4) and cloned into a Golden Gate variant of the pMESy4 phage display vector (KF415192) using SapI to construct camelid nanobody libraries. After two rounds of selection against either RelA_*Ec*_ or Rel_*Ct*_—either in unbound state or in the presence of GDP or APCPP—we successfully isolated, after phage display selection^68^, a set of different candidate binders that were classified according to the sequences of the third complementarity determining region (CDR3, see Supplementary Fig. 1a).

### Growth assays on SMG (Serine, Methionine and Glycine) plates

The SMG plate assay was performed as described earlier^17^. The strains were grown overnight in liquid M63^53^-glucose minimal medium supplemented with 0.4% glucose, 5 μg/mL thiamine, 100 μg/mL each of L-Arginine, L-Histidine, L-Leucine, L-Threonine and 100 µg/mL ampicillin. Optionally, 0.5 mM IPTG was used to induce nanobody expression and 1 mM each of L-Serine, L-Methionine, and L-Glycine was added to repress growth of RelA-deficient strains. The cultures were adjusted to OD_600_ 0.5 and 10-fold dilutions were plated on solid M63, SMG and SMG-IPTG plates^52^ supplemented with 100 µg/mL ampicillin.

### Virulence assays in *G. mellonella*

For inoculation, *G. mellonella* larvae were incubated for 1 h at 4 °C before injection. Overnight culture of *E. coli* Ec156^54^ transformed with the vector expressing the nanobodies (Supplementary Table 4 contains the genetic information for the constructs used throughout the manuscript) was washed and diluted to a cell density equivalent to 100 CFU in 0.9 % NaCl, 10 µL and was injected into the bottom left proleg of each larva. Twenty larvae were inoculated per strain and incubated at 37 °C in the dark. Viability of the larvae was scored every 12 h.

### RSH enzymes purification

Overexpression and purification of *E. coli* RelA and *C. tepidum* Rel were performed as described earlier^19,69^. To purify Rel_*Ct*_^NTD^ and variants, clarified cell lysate of N-terminally His-TEV-tagged proteins was loaded onto a HisTrap HP 1 mL column (GE Healthcare) pre-equilibrated with 5 column volumes of the binding buffer (50 mM Hepes pH 7.5, 500 mM KCl, 500 mM NaCl, 10 mM MgCl_2_, 1 mM TCEP and 0.002 % mellitic acid), 1 pastil of protease inhibitors cocktail (Roche)), respectively. A washing step with binding buffer preceded elution of the protein by a linear gradient of 0–500 mM imidazole. Elution fractions were pooled for size exclusion chromatography with a HiLoad 16/600 Superdex 200 PG column (GE Healthcare) pre-equilibrated with the respective binding buffer. Purified truncates were treated with TEV-protease in a 1:100 molar ratio, overnight, at room temperature to remove the His-tag. The protease and cleaved tag were removed by reverse IMAC using a TALON gravity flow column, followed by SEC to the protein buffer. The proteins were concentrated to an appropriate concentration using ultrafiltration units with 30 000 Da cut-off (Amicon Ultra, Merck Milipore). Purity of the samples’ preparation was assessed spectrophotometrically and by SDS-PAGE. All the RSH constructs of recombinant WT enzymes and mutants were generated by GenScript (https://www.genscript.com).

### Purification of nanobodies

In all cases, nanobodies were produced in *E. coli* WK6 cells^40^ transformed with the pMesy4 plasmid carrying the Nb-genes His-tagged at the C-terminus. Cells were grown in TB broth, and expression was induced with 500 μM IPTG overnight at 28 °C. Periplasmatic extraction of the nanobodies was done by osmotic shock, as previously described^68^. The His-tagged Nbs were purified using a His-trap Ni-NTA IMAC-column (Cytiva) on an AKTA pure FPLC-system at 8 °C in 25 mM HEPES 7.5; 250 mM NaCl; 1 mM TCEP. Elution of the nanobodies was performed by applying a gradient of imidazole (0–500 mM). Ni-NTA IMAC purification was followed by buffer exchange on a SEC column (Superdex75 increase 10/300 GL, Cytiva) to HEPES 7.5, 250 mM NaCl; 1 mM TCEP. Sample purity was assessed spectrophotometrically and by SDS-PAGE.

### Isothermal titration calorimetry (ITC)

All titrations were performed in triplicate with an Affinity-ITC (TA instruments) at 15 °C using a constant titration volume of 2 μL. Samples were incubated and degassed for 10 min prior to the ITC measurement. Nucleotide stocks of Adenosine-5’-[(α,β)-methyleno]triphosphate (APCPP), ppGpp, pppGpp (Jena Biosciences) and GDP (Sigma Aldrich) of 650–670 μM were diluted in 50 mM HEPES pH 7.5; 500 mM KCl; 500 mM; NaCl; 10 mM MgCl_2_; 1 mM TCEP; 0.002 % mellitic acid to a 16–22 fold excess relative to the protein in the ITC cell. The purified RSH-enzymes were concentrated by ultrafiltration (Amicon Ultra, 0.5 ml, 30 kDa, Merck Millipore) at 3000 g at 15 °C to a final concentration of 44–180 μM. All ITC measurements were performed using a constant stirring rate of 75 r.p.m. All data were processed and analyzed using the NanoAnalyse and Origin software packages; the reported mean K_*D*_ and SD of each titration are summarized in Supplementary Table 1.

### Enzymatic assays

Purified protein was incubated in HEPES Polymix buffer (as in refs. ^17,19,26,59^) with HEPES pH 7.5 and 0.5 mM TCEP to a final concentration of 0,25 µM. The Nbs (2.5 µM) or 70S ribosomal complexes (1.5 µM) (*E. coli*) were added prior to a 5 min incubation at room temperature. In case of deacylated tRNA (1 µM) (mix) and pppGpp-mediated (100 µM) stimulation of (p)ppGpp synthesis, these compounds were added after incubation with 70S ribosomal complexes for 5 min and incubated for a subsequent 5 min at room temperature. When monitoring the ppGpp-synthesis reaction, GDP was added to 1 mM final concentration to the mixture before incubation for 1 min at 40 °C. Synthesis of ppGpp was initiated by the addition of ATP (at 40 °C) to a final concentration of 1 mM. When monitoring the ppGpp-hydrolysis reaction, the reaction mix was incubated at 40 °C for 1 min before adding the substrate (ppGpp) at a final concentration of 1 mM. The reaction mixtures were incubated in a mixing heat block at 40 °C under rigorous shaking at 600 r.p.m. Quenching of the enzymatic reactions was achieved by the addition of formic acid (10 %). The reaction mixes were stored on ice and centrifuged for 1 min at 4 °C before analysis by means of HPLC.

HPLC was performed on a Shimadzu i-series device using an ACE-equivalent C18 HPLC column (3 µM, 30 × 4.6 mm, Avantor). Nucleotides were separated by reverse phase applying a gradient (0-100 %) of buffer B (100 mM K phosphate, pH 6.4, 5 mM tetrabutyl ammonium bromide (TBAB), 15 % acetonitrile) to buffer A (10 mM K phosphate buffer, pH 6.4, 5 mM TBAB, 0.1 % acetonitrile) at a flow rate of 0.8 mL/min for 10 min. The nucleotides were monitored by absorbance at 254 nm, and the concentration of GDP and ppGpp was derived from calibration curves using the surface area of the respective peaks. All measurements were analyzed using the Labanalysis software. Turnovers were obtained from linear regression of the GDP, or ppGpp synthesized in time. All experiments were performed in triplicate.

### Crystallization of Rel_Ct_^NTD^/Nb898-complex

Crystals of Rel_*Ct*_^NTD^ in complex with GDP and APCPP were obtained by incubating Rel_*Ct*_^NTD^ at 10 mg/mL with 10 mM (final concentration) of the nucleotides. Crystals were obtained in the F8 condition of the LMB crystallization screen, 18 % w/v PEG 4000, 0.1 M Tris pH 9.0 and 0.3 M Sodium acetate trihydrate (Molecular Dimensions), at 20 °C using the sitting-drop vapor-diffusion technique with Swissci (MRC) 96-well 2-drop UVP sitting-drop plates. The 200 nL drops of 1:1 crystallization solution/protein sample were deposited under humidity-controlled conditions using the Mosquito robotic system (TTP Labtech). The drops were equilibrated to 80 μL of the crystallization solution on the plate. For data collection, the crystals were directly harvested from the screening plate.

To obtain crystals of Rel_*Ct*_^NTD^ in complex with Nb898, the nanobody was added in a 5-fold molar excess to purified Rel_*Ct*_^NTD^ and incubated for 5 min at room temperature. SEC on an Åkta pure FPLC system at 8 °C with a Superdex 200 increase 10/300 GL column (Cytiva) equilibrated with the protein buffer at 2% glycerol was performed on the mixture to isolate the protein complex. Formation of the complex was verified on denaturing SDS-PAGE. The protein complex was concentrated by ultrafiltration using a 3000 Da cut-off (Amicon Ultra, Merck Milipore) to 14 mg/mL as estimated by OD_280nm_ (on a NanoDrop microvolume spectrophotometer, Thermo-Fisher Scientific) using theoretical extinction coefficients. Crystals were obtained in D9 condition of the LMB crystallization screen, 27% w/v PEG 3350, 0.1 M Bis-Tris propane, pH 7.0 and 0.2 M Li_2_SO4 (Molecular Dimensions) at 20 °C, using the sitting-drop vapor-diffusion technique with Swissci (MRC) 96-well 2-drop UVP sitting-drop plates. The 200 nL drops of 1:1 crystallization solution/protein sample were deposited under humidity-controlled conditions using the Mosquito robotic system (TTP Labtech). The drops were equilibrated to 80 μL of the crystallization solution on the plate. For data collection, the crystals were directly harvested from the screening plate.

### Structure determination

X-ray diffraction data of crystals of Rel_*Ct*_^NTD^:Nb898 were collected at the PX2 beamline of the Soleil synchrotron (Gif-sur-Yvette, France) on an Eiger detector. Prior to data collection, the crystals were transferred to a cryoprotectant solution containing 38% of 2-Methyl-1,3-propanediol (MPD) and flash-cooled in liquid nitrogen. The data were processed using XDS suite^37^ and scaled with Aimless. The anisotropic analysis of the diffraction data suggested a resolution of 3.15 Å (with diffraction limits of 3.39 Å in a*, 4.99 Å in b* and 2.73 Å in c*). The structure was solved by molecular replacement performed with Phaser^38^ using the coordinates of nanobody T4^40^ (PDB ID: 6GJS) after removing the CDR regions and the coordinates of Rel_*Tt*_^NTD^. Initial automated model building was performed with Buccaneer^42^, which partially completed the Nb898 and was further improved with the MR-Rosetta suite from the Phenix package^43^. After several iterations of manual building with Coot^41^ and maximum likelihood refinement as implemented in Buster/TNT^44^, the model was refined to R/Rfree of 0.243/0.283.

In the case of the Rel_*Ct*_^NTD^:GDP:APCPP complex, X-ray diffraction data were collected at the PX2 beamline of the Soleil synchrotron (Gif-sur-Yvette, France) on an Eiger detector. Crystals were cryo-protected in 20% glycerol and 2% DMSO and flash-cooled in liquid nitrogen prior to data collection. The data were processed using XDS suite^37^ and scaled with Aimless. Anisotropic analysis of the diffraction data suggested a resolution of 2.65 Å (with diffraction limits of 3.07 Å in a*, 2.65 Å in b* and 2.80 Å in c*). The structure was solved by molecular replacement performed with Phaser^38^ using the coordinates of the separated HD and SYNTH domains of Rel_*Ct*_^NTD^ (from the Rel_*Ct*_^NTD^-Nb898 complex). Automated model building was performed using the MR-Rosetta suite from the Phenix package^43^. After several iterations of manual building with Coot^41^ and maximum likelihood refinement as implemented in Buster/TNT^44^, the model was refined to R/Rfree of 0.187/0.228. The X-ray data collection and refinement statistics of both datasets are summarized in Supplementary Table 3.

### Hydrogen-deuterium exchange mass spectrometry

HDX-MS experiments were performed on an HDX platform composed of a Synapt G2-Si mass spectrometer (Waters Corporation) connected to a nanoACQUITY ultraperformance liquid chromatography (UPLC) system, as described^70^. Samples of Rel_*Ct*_^NTD^, Nb898, Nb585 the Rel_*Ct*_^NTD^:Nb898 and Rel_*Ct*_^NTD^:Nb585 complexes were prepared at a concentration of 50 to 70 μM. For each experiment, 5 μL of sample was incubated for 0.5, 5, or 60 min in 95 μL of labeling buffer L [50 mM HEPES, 500 mM KCl, 500 mM NaCl, 2 mM MgCl_2_, 1 mM TCEP, and 0.002% mellitic acid (pD 7.5)] at 20 °C. The nondeuterated reference points were prepared by replacing buffer L with equilibration buffer E [50 mM Hepes, 500 mM KCl, 500 mM NaCl, 2 mM MgCl_2_, 1 mM TCEP, and 0.002% mellitic acid (pH 7.5)]. After labeling, the samples are quenched by mixing with 100 μL of prechilled quench buffer Q [1.2% formic acid (pH 2.4)]. 70 μL of the quenched samples were directly transferred to the Enzymate BEH Pepsin Column (Waters Corporation) at 200 μL/min and at 20 °C with a pressure of 8.5 kpsi. Peptic peptides were trapped for 3 min on an ACQUITY UPLC BEH C18 VanGuard precolumn (Waters Corporation) at a flow rate of 200 μL/min in water [0.1% formic acid in HPLC water (pH 2.5)] before being eluted to an ACQUITY UPLC BEH C18 column for chromatographic separation. Separation was performed with a linear gradient buffer (7 to 40% gradient of 0.1% formic acid in acetonitrile) at a flow rate of 40 μL/min. Identification of peptides and deuteration uptake analysis was performed on the Synapt G2Si in (ESI + )—HDMSE mode (Waters Corporation). Leucine enkephalin was applied for mass accuracy correction, and sodium iodide was used as calibration for the mass spectrometer. HDMSE data were collected by a 20- to 30-V transfer collision energy ramp. The pepsin column was washed between injections using pepsin wash buffer [1.5 M Gu-HCl, 4% (v/v) acetonitrile, and 0.8% (v/v) formic acid]. A blank run was performed between each sample to prevent significant peptide carryover. Optimized peptide identification and peptide coverage for all samples were performed from undeuterated controls (five replicates). All deuterium time points were performed in triplicate (All HDX-MS parameters are shown in Supplementary Table 2).

### Reporting summary

Further information on research design is available in the Nature Portfolio Reporting Summary linked to this article.

## Supplementary information

Supplementary Information

Reporting Summary

Transparent Peer Review file

## Source data

Source Data

## Data Availability

Structural data from this study are available from the Protein Data Bank (PDB) under accessions 9S6K and 9S6L. HDX raw data can be accessed at the PRoteomics IDEntifications (PRIDE) Archive database under the accession PXD067451. The characteristics and profiles of each nanobody are available in the Nanosaurus database (https://nanosaurus.org) under accession codes SD-QXVA (Nb94), SD-NP51 (Nb585), SD-UMV2 (Nb898) and SD-G7X6 (Nb96). PDB codes of previously published structures used in this study are 6S2U, 8ACU and 6GJS. Materials, including strains and plasmids, are available upon request. Source data are provided as a Source Data file. Source data are provided with this paper.
