# Supplementary material for: Nanobody-mediated control of long RSH Rel and RelA catalysis by restriction of their conformational landscape
Source: Nat Commun. 2026 May 13;17:6368. doi: 10.1038/s41467-026-73059-3 (PMC13376610; doi:10.1038/s41467-026-73059-3)
Supplement: Supplementary file 1 — Supplementary Information [file 41467_2026_73059_MOESM1_ESM.pdf]

Supplementary Information

Supplementary Figures

Supplementary Fig. 1. The TGS H432 directly coordinates Nb94 and Nb96. (a) Multiple sequence alignment of the nanobodies Nb94, Nb96 and the control Nb120 (each CDR region is boxed and labeled). Details of the binding interface of Nb94 **(b)** and Nb96 **(c)** as predicted by AlphaFold. CDR regions involved in binding are indicated by a black arrow. Interface residues contributed by the nanobodies and involved the coordination of H432 are shown in italic. From the prediction it is clear that the introduction of a negative charge via the H432E substitution will significantly disturb the binding interface. ITC titrations of Nb94 **(d)** and Nb96 **(e)** into RelA$_{Ec}^{H432E}$. The lack of binding observed strongly supports the predicted mode of binding.

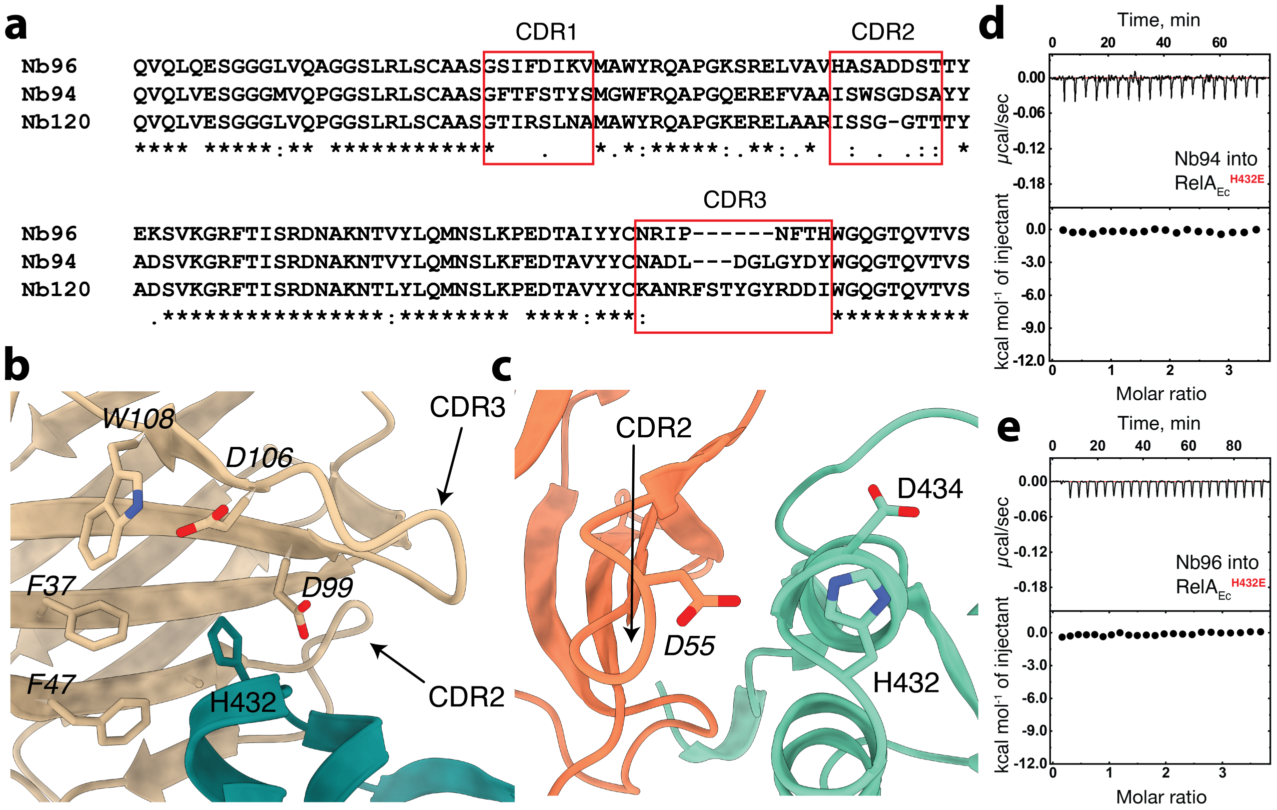

Supplementary Fig. 2. Nb898 and Nb585 have notably different CDR regions. Sequence comparison of Rel$_{Ct}$ binders, Nb898 and Nb585. The comparison shows the strong differences in the observed complementarity determining region (CDR) that defined the contrasting activities of these nanobodies. Each CDR region is labeled in the figure.

```
                                           CDR1                              CDR2
Nb898    QVQLQESGGGLVQAGGSLRLSCAASGSIESINAMAWYRQAPGKPRELVAVIAKSDGSTTY
Nb585    QVQLQESGGGLVQAGGSLRLSCAASGFTFEDYTIGWFRQAPGKEREGVSLISNSDGFTSY
         **************************   .  ::.*:****** ** *::*::*** *:*

                                                        CDR3
Nb898    AVPVKGRFTISRDDAKNTVYLQMNSLKPEDTAIYYCNKI----------PNFTYWGQGTQ
Nb585    ADSVKGQFTISSENAKNTVYLQMDNLKPEDTAVYYCAATEVLAHLMWRPSNWGYWGQGTQ
         *  ***:**** ::*********:.*******:***              *: *******

Nb898    VTV
Nb585    VTV
         ***
```

Supplementary Fig. 3. HDX-MS analysis of the Rel$_{Ct}^{NTD}$-Nb898 interface. Heatmaps representing the hydrogen/deuterium exchange (HDX) at different times of Rel$_{Ct}^{NTD}$ (top), Rel$_{Ct}^{NTD}$-Nb898 complex **(a)** and ΔHDX Rel$_{Ct}^{NTD}$: Rel$_{Ct}^{NTD}$-Nb898 complex **(b)**. The RFU scale shows the relative fractional uptake. In the bottom plot the ΔRFU represents the differences in exchange, with blue representing maximum protection and red maximum uptake. The residues predicted to be involved in the Rel$_{Ct}^{NTD}$-Nb898 binding interface (i.e those that show strong protection) are outlined by a dashed red line. **(c)** Bar plot representing differences in deuterium uptake between free- and Nb898 bound Rel, with each bar corresponding to a unique peptide from the peptide library (ordered from N- to C- terminus based on the first amino acid of each peptide). Light grey shades show the differential uptake s.d for each peptide. Differential uptake (Woods plot) upon Nb 898 binding (98% C.I., pvalue > 0.02) after incubation at room temperature in deuterated solvent for 30 sec **(d)**, 5 min **(e)**, or 60 min **(f)** for peptides from 116 to 127 and 30 sec **(g)**, 5 min **(h)**, or 60 min **(i)** for peptides from 65 to 77. Bars represent individual peptides. Bar length corresponds to the peptide size. Red coloured bars indicate statistically significant deprotected peptides, (highlighted region 118-127) and blue coloured bars indicate statistically significant protected peptides, (highlighted region 65-77). Source data can be accessed at the PRoteomics IDEntifications (PRIDE) Archive database under the accession PXD067451.

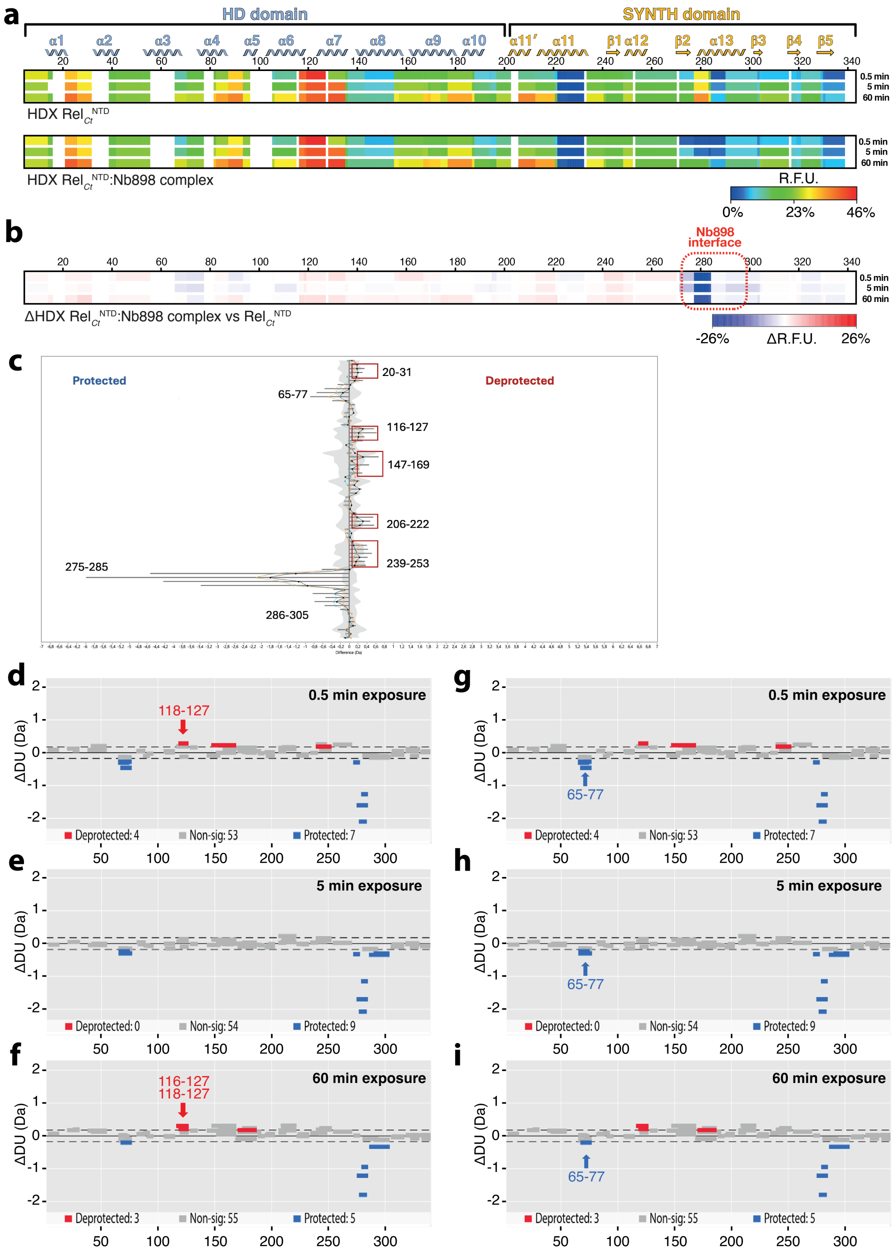

Supplementary Fig. 4. Structural characterisation of the Rel$_{Ct}$^{NTD}-Nb898 interface. Electron density maps contour at 1.0σ around the HD **(a)**, SYNTH **(b)**, Nb898 **(c)** and the RelCt-Nb898 complex interface, showing details of the side chains contributed by both proteins **(d)**. Interaction of the SYNTH domain of Rel with DarB (Rel$_{Bs}$-DarB, PDB ID 8ACU) **(e)** and Rel$_{Ct}$ with Nb898 (this work) **(f)**. These allosteric contacts with the SYNTH domain enhance the (p)ppGpp synthetase activity of Rel and n both cases α13 is the main contact point with additional interactions contributed by α11 and the active site G-loop. Simulated annealing composite omit maps at 1.0σ electron density map corresponding to the visible groups of GDP bound to Rel$_{Ct}$^{NTD}, 5'-pyrophosphate group of GDP in chain B **(g)**, chain C **(h)**, and chain D **(i)**.

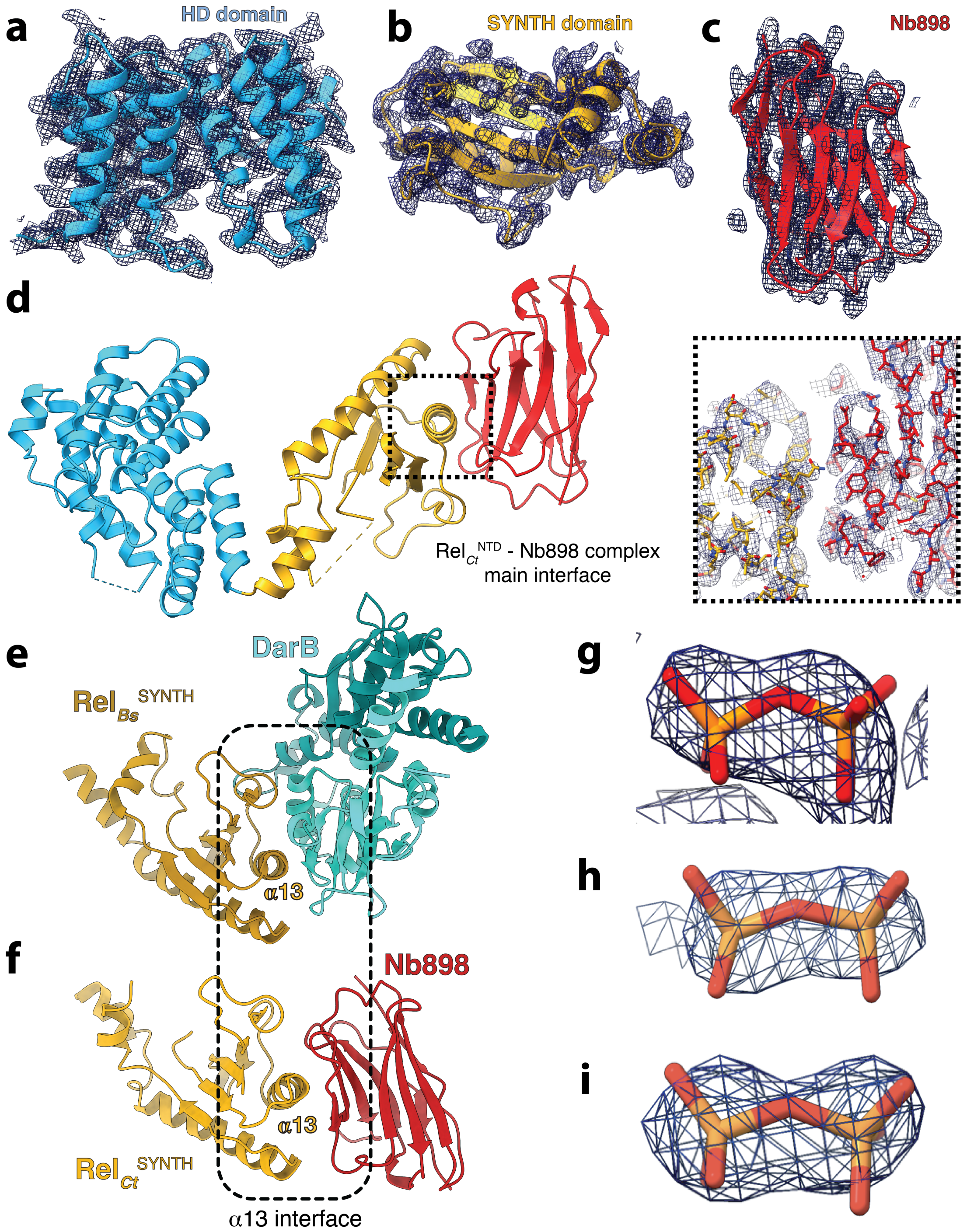

Supplementary Fig. 5. Nb585 stabilises a conformation that resembles the active HD state of Rel. **(a)** Structural superposition of Rel_{Ct}^{NTD} in the complex with Nb585, in the closed conformation predicted by Alphafold onto the Rel_{Tt}-ppGpp complex. The comparison supports the hypothesis that binding to Nb585 triggers a state that favours hydrolysis (illustrated by the presence of ppGpp in the HD site) and prevents synthesis (the SYNTH active site is largely occluded in this state). **(b)** Cartoon representation of the Alphafold predicted model of the Rel_{Ct}^{NTD}-Nb585 complex coloured as in Fig. 5c. The main contact interface is highlighted by a black square. **(c)** Details of Rel_{Ct}^{NTD}-Nb585 complex interface highlighting key residues involved in binding including D31 of Nb585 (in blue) that forms a slat bridge with R215 of Rel_{Ct}^{NTD} (in gold).

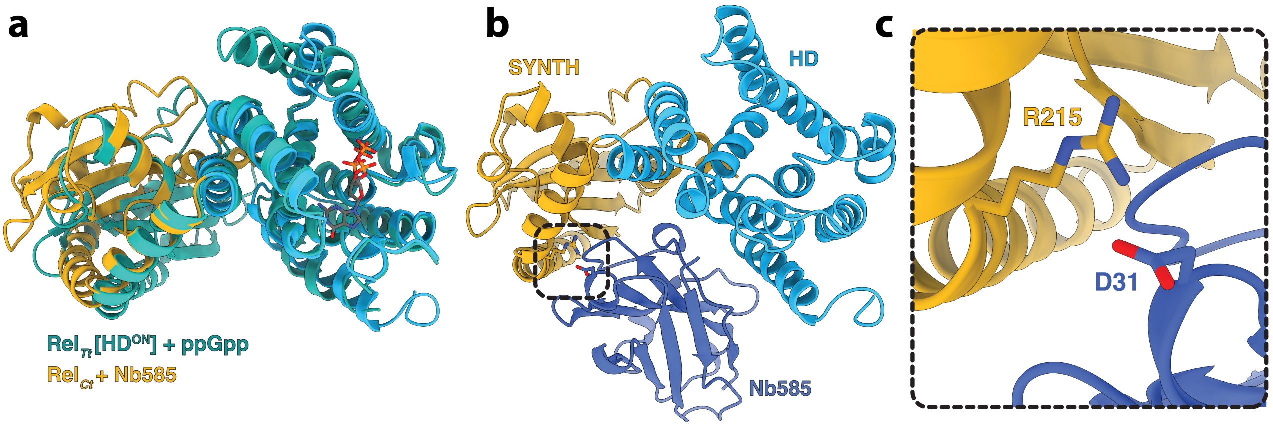

Supplementary Tables

Supplementary Table S1. Binding parameters determined by Isothermal Titration Calorimetry (ITC). Experimentally determined binding thermodynamic parameters from all ITC measurements (mean K_D and SD resulting from three titrations). N.D. stands for 'not detectable'. All the titrations are background-subtracted.

Titrations	K_D (µM)	ΔH (kcal/mol)	$-T\Delta S$ (kcal/mol)	ΔG (kcal/mol)	Molar Ratio (n)
Nb94 into RelA$_{Ec}$	0.013±0.001	-10.1±0.1	-0.5±0.1	-10.6±0.01	0.99
Nb96 into RelA$_{Ec}$	0.080±0.004	-13.5±0.1	4.0±0.1	-9.5±0.01	0.95
Nb120 into RelA$_{Ec}$	N.D.	-	-	-	-
Nb94 into RelA$_{Ec}^{H432E}$	N.D.	-	-	-	-
Nb96 into RelA$_{Ec}^{H432E}$	N.D.	-	-	-	-
Nb898 into Rel$_{Ct}$	0.186±0.005	-6.8±0.2	-2.8±0.1	-9.6±0.02	0.9
Nb585 into Rel$_{Ct}$	0.021±0.001	-8.5±0.1	-1.7±0.1	-10.2±0.01	0.92
Nb898 into Rel$_{Ct}^{NTD}$	0.19±0.01	-2.3±0.1	-6.8±0.3	-9.2±0.02	1.1
Nb898 into Rel$_{Ct}^{K280A}$	2.20±0.5	-9.5±0.9	1.8±0.1	-7.7±0.1	0.90
Nb898 into Rel$_{Ct}^{D283A}$	0.20±0.005	-7.2±0.3	-1.9±0.2	-9.1±0.02	0.90
Nb898 into Rel$_{Ct}^{F285A}$	N.D.	-	-	-	-
Nb898 into Rel$_{Ct}^{A286K}$	12.0±1	-13.4±0.8	6.7±0.5	-6.7±0.1	0.97
Nb898 into Rel$_{Ct}^{Y290A}$	1.10±0.8	-11.1±0.3	3.0±0.2	-8.1±0.1	0.91
Nb585 into Rel$_{Ct}^{R215A}$	0.60±0.1	-8.8±0.1	0.7±0.2	-8.1±0.02	0.8
APCPP into Rel$_{Ct}$	37.9±2	-5.9	0.1	-5.8	0.97
APCPP into Rel$_{Ct}$:Nb898	28.9±1	-7.4	1.4	-6.0	0.92
GDP into Rel$_{Ct}$	N.D.	-	-	-	-
GDP into Rel$_{Ct}$:APCPP	69.8±5	-18.8±1	13.3±0.8	-5.5±0.3	0.90
GDP into Rel$_{Ct}$:Nb898	7.10±0.8	11.3±0.3	-18.1±0.8	-6.8±0.1	0.92

Supplementary Table S2. Hydrogen Deuterium eXchange coupled with Mass Spectrometry (HDX-MS).

	**State A: Rel$_{Ct}$**	**State B: Rel$_{Ct}$ + Nb898**	**State C: Rel$_{Ct}$ + Nb585**
HDX time course (min)	0, 0.5, 5, 60	0, 0.5, 5, 60	0, 0.5, 5, 60
HDX control samples	None	None	None
Back-exchange (mean / IQR)	N/A	N/A	N/A
# of Peptides	70		
Sequence coverage (%)	88.18	88.18	88.18
Average peptide length / redundancy	11.83 / 2.71	11.83 / 2.71	11.83 / 2.71
Replicates (technical)	3	3	3
Repeatability: (average SD)	0.0325	0.0361	0.0400

Supplementary Table S3. X-ray data collection and processing. The $CC_{1/2}$ criterion was used to determine the resolution range. Values for the outer shell are given in parentheses.

	**Rel$_{Ct}^{NTD}$: Nb898 complex**	**Rel$_{Ct}^{NTD}$: GDP : APCPP complex**
**Crystallization condition**	29 % w/v PEG 6000 0.2 M Sodium citrate	27 % w/v PEG 3350 0.1 M Bis-Tris propane 0.2 M Lithium sulfate
**Data Collection Statistics**		
Diffraction source	Soleil PX2	Soleil PX2
Wavelength (Å)	0.9762	0.9801
Temperature (K)	100	100
Detector	Dectris PILATUS 6M	Dectris Eiger
Detector distance (mm)	602.7	342.5
Space group	C2	$P12_11$
a, b, c (Å)	197.43 88.30 62.52	92.63 92.31 122.29
α, β, γ (°)	90.00 101.88 90.00	90.00 94.80 90.00
Resolution range (Å)	80.309 - 3.15 (3.37 – 3.15)	50.85 - 2.65 (2.89 - 2.65)
Total No. of reflections	46141 (1971)	248789 (11760)
No. of unique reflections	11244 (562)	46040 (2302)
Completeness (%)	91.0 (87.5)	93.5 (53.5)
Redundancy	4.1 (3.5)	5.4 (5.1)
$\langle I/\sigma(I) \rangle$	3.8 (1.6)	9.0 (1.1)
R_{merge}	0.626 (1.657)	0.15 (1.33)
CC1/2	0.822 (0.387)	0.994 (0.411)
Overall *B* factor / Wilson plot (Å^2)	38.32	63.8
**Refinement Statistics**		
R-factor (%)	27.8	21.6
R_{free}-factor (%)	30.6	23.5
Ramachandran profile (%)		
Core	97.1	98.3
Allowed	2.94	1.7
Outliers	0.0	0.0
R.m.s. deviations		
Bond lengths (Å)	0.01	0.013
Bond angles (°)	1.29	1.60
Number of atoms	6261	11065
Macromolecules	6259	10679
Solvent	0	204
Ligands	-	171
Metals	2	11
B-factors (Å^2)		
All atoms	37.21	70.4
Macromolecules	37.21	70.5
Solvent atoms	-	56.6
Metals	-	67.35
Nucleotides	-	91.05
Other atoms	37.38	66.33
PDB ID	9S6K	9S6L

Supplementary Table S4. Strains and plasmids used in this study.

Plasmids	Description	Reference
pET24d-His10-SUMO kmR	Expression vector for SUMO-tagged protein	Laboratory stock
pET24d-His10-SUMO-relct kmR	Expression vector for SUMO-tagged *C. tepidum* Rel	This study
pET24d-His10-SUMO-relct_K280A kmR	Expression vector for SUMO-tagged R215A-substituted *C. tepidum* Rel	This study
pET24d-His10-SUMO-relct_K280A kmR	Expression vector for SUMO-tagged K280A-substituted *C. tepidum* Rel	This study
pET24d-His10-SUMO-relct_D283A kmR	Expression vector for SUMO-tagged D283A-substituted *C. tepidum* Rel	This study
pET24d-His10-SUMO-relct_F285A kmR	Expression vector for SUMO-tagged F285A-substituted *C. tepidum* Rel	This study
pET24d-His10-SUMO-relct_A286K kmR	Expression vector for SUMO-tagged A286K-substituted *C. tepidum* Rel	This study
pET24d-His10-SUMO-relct_Y290A kmR	Expression vector for SUMO-tagged Y290A-substituted *C. tepidum* Rel	This study
pET24d-His10-SUMO-relct_NTD kmR	Expression vector for SUMO-tagged NTD (catalytic) region of *C. tepidum* Rel	This study
pET24d-His10-SUMO-relA (residues 1–744) kmR	Expression vector for SUMO-tagged *E. coli* RelA	Turnbull et al., 2019 doi: 10.3389/fmicb.2019.01966

pET24d-His10-SUMO-relA_H432A (residues 1–744) kmR	Expression vector for SUMO-tagged H432E-substituted *E. coli* RelA	Turnbull et al., 2019 doi: 10.3389/fmicb.2019.01966
pMesy4_Nb94-His	Expression vector for C-term-His-tagged Nb94	This study
pMesy4_Nb96-His	Expression vector for C-term-His-tagged Nb96	This study
pMesy4_Nb898-His	Expression vector for C-term-His-tagged Nb898	This study
pMesy4_Nb585-His	Expression vector for C-term-His-tagged Nb585	This study
Strains		
E. coli BL21 DE3	B F^- ompT *gal dcm lon hsdSB*(rB^-mB^-) λ(DE3 [*lacI lacUV5-T7p07 ind1 sam7 nin5*]) [$malB^+$]$_{K-12}$(λ^S)	Laboratory stock
E. coli WK6 cells	F' lacIq Δ(lacZ)M15 proA+B+ Δ(lac-proAB) galE rpsL	Laboratory stock
E. coli Ec156	WT	Hertz F.B. et al. 2025
Primers		
call001	GTCCTGGCTGCTCTTCTACAAGG	Desmyter, A. et al. (1996)
call002	GGTACGTGCTGTTGAACTGTTCC	Desmyter, A. et al. (1996)
229	CCTTGAGCTCTTCGGCACAGGTGCAGCTGGTGGAGTCTGG	This study
230	AGGACTGCTCTTCCACTGGAGACGGTGACCTGGGT	This study
