# Supplementary material for: Nanobody-mediated control of long RSH Rel and RelA catalysis by restriction of their conformational landscape
Source: Nat Commun. 2026 May 13;17:6368. doi: 10.1038/s41467-026-73059-3 (PMC13376610; doi:10.1038/s41467-026-73059-3)
Supplement: Supplementary file 2 — Reporting Summary [file 41467_2026_73059_MOESM2_ESM.pdf]

nature portfolio

Corresponding author(s): Abel Garcia-Pino

Last updated by author(s): Apr 19, 2026

Reporting Summary

Nature Portfolio wishes to improve the reproducibility of the work that we publish. This form provides structure for consistency and transparency in reporting. For further information on Nature Portfolio policies, see our Editorial Policies and the Editorial Policy Checklist.

Statistics

For all statistical analyses, confirm that the following items are present in the figure legend, table legend, main text, or Methods section.

n/a	Confirmed	
☐	☒	The exact sample size (*n*) for each experimental group/condition, given as a discrete number and unit of measurement
☐	☒	A statement on whether measurements were taken from distinct samples or whether the same sample was measured repeatedly
☒	☐	The statistical test(s) used AND whether they are one- or two-sided<br>*Only common tests should be described solely by name; describe more complex techniques in the Methods section.*
☒	☐	A description of all covariates tested
☒	☐	A description of any assumptions or corrections, such as tests of normality and adjustment for multiple comparisons
☒	☐	A full description of the statistical parameters including central tendency (e.g. means) or other basic estimates (e.g. regression coefficient) AND variation (e.g. standard deviation) or associated estimates of uncertainty (e.g. confidence intervals)
☒	☐	For null hypothesis testing, the test statistic (e.g. *F*, *t*, *r*) with confidence intervals, effect sizes, degrees of freedom and *P* value noted<br>*Give P values as exact values whenever suitable.*
☒	☐	For Bayesian analysis, information on the choice of priors and Markov chain Monte Carlo settings
☒	☐	For hierarchical and complex designs, identification of the appropriate level for tests and full reporting of outcomes
☒	☐	Estimates of effect sizes (e.g. Cohen's *d*, Pearson's *r*), indicating how they were calculated

Our web collection on statistics for biologists contains articles on many of the points above.

Software and code

Policy information about availability of computer code

Data collection	We did not generate any new software code for our work.
Data analysis	All the softwares used for the analysis of our data are described in the Material and Methods of the paper. They consists of Microcal Origin (v.20) and NanoAnalyze (3.12.05) for the analysis of all the calorimetric data. The crystallographic data were analyzed with the CCP4 (v. 8.0), PHENIX (v. 1.2), autoPROC (v. 20230217) and autoBUSTER (v. 20250717) suites.

For manuscripts utilizing custom algorithms or software that are central to the research but not yet described in published literature, software must be made available to editors and reviewers. We strongly encourage code deposition in a community repository (e.g. GitHub). See the Nature Portfolio guidelines for submitting code & software for further information.

Data

Policy information about availability of data

All manuscripts must include a data availability statement. This statement should provide the following information, where applicable:

- Accession codes, unique identifiers, or web links for publicly available datasets
- A description of any restrictions on data availability
- For clinical datasets or third party data, please ensure that the statement adheres to our policy

We described in our paper 2 crystal structures. The coordinates of these structures have been deposited in the PDB under the accession codes: 9S6K, and 9S6L. The mass spectrometry (HDX-MS) data have been deposited to the ProteomeXchange Consortium via the PRIDE partner repository with the dataset identifier PXD067451. All data needed to evaluate the conclusions are present in the paper and/or the Methods. Additional data related to this paper may be requested from

the authors.

Research involving human participants, their data, or biological material

Policy information about studies with human participants or human data. See also policy information about sex, gender (identity/presentation), and sexual orientation and race, ethnicity and racism.

Reporting on sex and gender	not relevant
Reporting on race, ethnicity, or other socially relevant groupings	not relevant
Population characteristics	not relevant
Recruitment	not relevant
Ethics oversight	not relevant

Note that full information on the approval of the study protocol must also be provided in the manuscript.

Field-specific reporting

Please select the one below that is the best fit for your research. If you are not sure, read the appropriate sections before making your selection.

[x] Life sciences [ ] Behavioural & social sciences [ ] Ecological, evolutionary & environmental sciences

For a reference copy of the document with all sections, see nature.com/documents/nr-reporting-summary-flat.pdf

Life sciences study design

All studies must disclose on these points even when the disclosure is negative.

Sample size	not relevant, our work does not include sampling large populations.
Data exclusions	no data were excluded from the analysis.
Replication	all replicates were taken into account when analyzing and presenting the data.
Randomization	not relevant
Blinding	not relevant

Reporting for specific materials, systems and methods

We require information from authors about some types of materials, experimental systems and methods used in many studies. Here, indicate whether each material, system or method listed is relevant to your study. If you are not sure if a list item applies to your research, read the appropriate section before selecting a response.

Materials & experimental systems

n/a	Involved in the study	
[ ]	[x]	Antibodies
[x]	[ ]	Eukaryotic cell lines
[x]	[ ]	Palaeontology and archaeology
[ ]	[x]	Animals and other organisms
[x]	[ ]	Clinical data
[x]	[ ]	Dual use research of concern
[x]	[ ]	Plants

Methods

n/a	Involved in the study	
[x]	[ ]	ChIP-seq
[x]	[ ]	Flow cytometry
[x]	[ ]	MRI-based neuroimaging

Antibodies

Antibodies used	Nb94, Nb96, Nb898, Nb585
Validation	We used nanobodies obtained from camelid antibodies that were generated in the current work. The sequence and specificity of these nanobodies were validated as part of this work.

Animals and other research organisms

Policy information about studies involving animals; ARRIVE guidelines recommended for reporting animal research, and Sex and Gender in Research

Laboratory animals	Lama glama (used to generate nanobodies)
Wild animals	not relevant
Reporting on sex	not relevant
Field-collected samples	not relevant
Ethics oversight	To generate camelid antibodies, llamas were immunized and blood samples were collected in full compliance with European legislation (EU Directive 2010/63/EU) and the Belgian Royal Decree of 29 May 2013 concerning the protection of laboratory animals. The animals were housed at a licensed facility (accreditation number LA 1700601) recognized by the Belgian competent authorities. Animal welfare was continuously monitored by an accredited veterinarian. All procedures, including immunization and blood collection, were reviewed and approved by the local Animal Ethics Committee.

Note that full information on the approval of the study protocol must also be provided in the manuscript.

Plants

Seed stocks	not relevant
Novel plant genotypes	not relevant
Authentication	not relevant
