# Supplementary material for: Nanobody-mediated control of long RSH Rel and RelA catalysis by restriction of their conformational landscape
Source: Nat Commun. 2026 May 13;17:6368. doi: 10.1038/s41467-026-73059-3 (PMC13376610; doi:10.1038/s41467-026-73059-3)
Supplement: Supplementary file 3 — Transparent Peer Review file [file 41467_2026_73059_MOESM3_ESM.pdf]

nature portfolio

Peer Review File

Nanobody-mediated control of long RSH Rel and RelA catalysis by restriction of their conformational landscape

Corresponding Author: Professor Abel Garcia-Pino

This file contains all reviewer reports in order by version, followed by all author rebuttals in order by version.

Version 0:

Reviewer comments:

Reviewer #1

(Remarks to the Author)
In the current manuscript „Nanobody-mediated control of long RSH Rel and RelA catalysis by restriction of their conformational landscape“ by Van Nerom et al. (NCOMMS-25-68425) the labs of Vasili Hauryliuk and Abel Garcia-Pino build on their previous works about RSH enzymes and conceive nanobodies effective against the primary (p)ppGpp enzyme Rel/RelA, including a rigorous analysis of their modes of action. As to my knowledge, the presented nanobodies are the first molecules acting allosterically on Rel activity with nanomolar affinities, whereas the handful of small molecule inhibitors described in literature act competitively with micromolar affinities. The description of these first-in-class nanobodies against Rel thus makes me quite enthusiastic about the paper.

In short, in the current study the authors raised nanobodies in camels that bind to the Escherichia coli RelA protein in vitro, impact E. coli growth upon overexpression in vivo and are also effective in an infection model. For further structural, biochemical and biophysical studies the authors switched to Rel from a different organism (Chlorobaculum tepidum, Ct) due to its better behaviour in vitro and provide mostly conclusive evidence on the modes of action of two distinct nanobodies raised against different states of the bifunctional RelCt protein. These results are put into context of ‚endogenous' Rel activity modulators, i.e., factors that govern Rel activity in bacteria.

The manuscript as a whole is well-written and describes the structural biology of Rel (and how it is impacted differently by the employed nanobodies) well even for the non-expert reader. The analyses in the study employ a timely set of methodology (X-ray crystallography and occasionally Alphafold-modelling, affinity and activity assays, hydrogen/deuterium exchange mass spectrometry) that is mostly well-executed and described.

Thematically, I do not see major blank pages in the presented study at hand. I could have only wished for more in vivo experimentation and application of the nanobodies with techniques other than their overexpression in bacterial host such as E. coli (experiments in Fig. 1). This would adress the most obvious next step dealing with pharmacology of the drugs, primarily their delivery. However, I do understand that this would eventually require an entire additional study that the authors are currently anyways working on at probably.

I can nevertheless and unfortunately not recommend publication of the current study right away due to some shortcomings in methodology related to the AMPCPP/GDP-bound RelCtNTD structure by X-ray crystallography and the mode how Nb898 affects Rel activity by HDX-MS. Both of these do not put into question the key findings of the study, i.e. generation of nanobodies impacting Rel activity, and as soon as these issues are adressed I strongly recommend publication of this study.

Major points

1) Assessment of the crystal structures (mainly AMPCPP/GDP-bound RelCtNTD)

The space groups assigned in the ‚Full wwPDB X-ray Structure Validation Report' are not coherent with the space groups mentioned in the ‚Supplementary Table S2. X-ray data collection and processing'. Also, in this table the completeness in the high resolution shell of the AMPCPP/GDP-bound structure seems very low. The authors would please double-check these.

The structure of the RelCtNTD : Nb898 complex seems fine within the scope of the limited resolution of 3.15 Angstrom.

However, related to the RelCtNTD : GDP : APCPP complex structure I see a potential issue in the Validation Report and the provided electron density maps for the GDP ligand:

As per the validation report the GDP is modelled entirely into the density in chains A and B (p. 31+33). Generally, the 0.7 rmsd cut-off for the 2Fo-DFc is very liberal and a cut-off of 1-1.5 would be more conservative (eventually set arbitrarily by the generating software of the Validation Report?). But even at this low 0.7 cut-off the GDP is poorly explained by density, in particular the guanine base. In chains C and D (p. 32+33) only a pyrophosphate (instead of GDP) is placed in the Validation Report. The density of the APCPP (APC) in contrast looks convincing in all four chains (p. 33-36). Inspection of the mtz files suggests a rather poor agreement of experimental density and the GDP modelling - the density for GDP looks deceptively flat to me instead of harboring some bulbs here and there (substitutions at base, ribose OH's and phoshphate, compare to APC density). In particular the guanine base exhibits almost no density at all although one would expect well-defined density for this particular entity given ist mode of coordination (π-stacking with a tyrosine provided by the G-loop).

The authors themselves mention a low occupancy of the GDP (l. 276f) and it may just be that the Validation Report does not properly reflect this – but I hope this isn't just HEPES (from the purification buffer) or a PEG fragment (from the crystallization conditions) bound to RelCtNTD : APCPP instead of GDP. An unbiased Fo-Fc map of GDP, which seems OK'ish to me by eye, is provided in SFig. 3e but without mention of neither chain ID nor the contour level. Please amend these.

→ I am convinced that GDP binds to APC-preloaded RelCtNTD (ITC data in Fig. 4f) and also admit that this SYNTH substrate-bound structure is not the major finding of the manuscript but nevertheless must urge the authors to check this particular dataset again thoroughly.

In conjunction with that the authors argue in ll. 292ff about the low GDP occupancy being in agreement with ITC results. I can't follow this argumentation given that APCPP and GDP were both present at 10 mM during crystallization (methods section), which should be absolutely sufficient given the ITC-determined two-digit micromolar Kd's.

2) Assessment of the HDX-MS datasets (mainly Nb898 binding to RelCt)

Unfortunately I could not assess the HDX-MS data in detail despite having received the reviewer login token to the PRIDE database entry. Reason is that I could not open the DynamX file despite using the very same version number (3.0) mentioned in the data analysis procedure of the entry. Opening the DynamX sessions stopped when reading the lock mass data; I expect the same problem would occur after publication of the dataset.

The authors should check this PRIDE entry itself for some inconsistencies and amend (e.g., it states CapRelSJ46 and Gp57 as search sequences, which appears to be a copy-paste error from a previous submission on TA systems; a FASTA and the PLGS output may also be provided).
The manuscript is currently lacking a HDX source data file (or similar) stating HDX-MS experiment's characteristics and HDX values (compare to the tables provided in the recommendations by Masson GR et al., Nat Methods 2019), which is critical for non-expert readers to assess the data. Such a dataset could have ruled out my concerns related tot he mode of action how Nb898 affects Rel activity:

SFig. 3 highlights a pronounced HDX change of Rel in presence of Nb898 at res. approx. 280, which is consistent with the binding site by the presented crystal structure. Then, two peptides are presented (Figs. 2e and f), which shall provide evidence for secondary effects of Nb898 besides the binding event.
- A) The differences exhibited by the 118-127 and 209-221 peptides are of very small magnitude, and if these fluctuations are considered significant, what is with other areas such as residues 20-35, 155-170, 240-250?
- B) I do not consider the changes exhibited in the peptides 118-127 and 209-221 as confident due to their dynamics. In 118-127 the behaviour is odd in that HDX gets lower with increasing deuteration time suggesting a high level of experimental fluctuation for this seemingly disordered (by HDX progression over time) region. In 209-221 the red curve with Nb898 is just shifted to higher uptake values by a small margin, which can easily come from divering peptide ion intensities or inconsistent assignments of undeuterated ion clusters.

→ During preparation of the HDX-MS data in above-mentioned format they could check the two areas in question again for, e.g., more redundant peptides, to substantiate the claims on Nb898 action on Rel activity.

Minor points
• Some typos, e.g.:
- l. 333: Sentence/argument is not finished
- l. 339: missing an ATP „binding“
- l. 756: space missing between 20% and glycerol
- SI table 3: „subsituted“ throughout
→ These are just the one's I spotted when reading. The authors will certainly find some more and amend these.

• I am missing a (short) description of cloning methodology (incl. a supplementary table with oligos)

• Can the authors comment in the manuscript on the endergonic ITC curve of GDP into Nb898-bound RelCt in Fig. 4g? This is just unexpected (compare to GDP into APCPP-bound RelCt in Fig. 4f) and could indicate more action going on in the area of the G-loop, in which's close vicinity Nb898 is also implicated to bind.

• ITC methodology (ll. 705ff):
- l. 709 → I never saw nucleotides being dissolved at such high concentrations. Are you sure or was it actually micromolar?
- l. 713 → I assume these were the final concentrations of the prep. Concentration of Rel in the cell should be stated as well. Another column in Supp. Table 1 would be a good fit unleast you don't want to mention this in each ITC-depicting figure caption.

• Crystallization (ll. 718ff):
- Would be nice if authors could explicit the crystallization conditions in which crystal were obtained instead of stating the well of the screen alone.

• The separately-supplied preparation of the camelid nanobodies should be integrated into the materials & methods section.

Reviewer #2

(Remarks to the Author)
It is well established that Rel/SpoT homolog (RSH) enzymes, the central regulators of the bacterial stringent response, adopt distinct conformational states to carry out either their synthetase or hydrolase functions. In this study, Nerom and colleagues introduce a novel set of camelid-derived nanobodies capable of locking long RSH enzymes into these discrete functional states. By immunizing llamas with RSH proteins, the authors generated nanobodies that selectively stabilize alternative conformations, effectively enabling constitutive activation or repression of enzymatic activity—independent of ribosomal regulation.
Through an integrated approach combining structural prediction, high-resolution structural analysis, biochemical assays, and microbiological techniques, the authors mapped the nanobody binding sites and elucidated their mechanisms of action. Notably, they demonstrated that overexpression of these nanobodies in E. coli reduced bacterial virulence in a Galleria mellonella (wax moth) infection model, highlighting the potential of nanobodies as tools for modulating bacterial pathogenicity and informing antimicrobial strategies.
This work presents an interesting case for the use of nanobodies in RSH research, both as molecular probes to dissect regulatory mechanisms and as potential scaffolds for therapeutic development.

Major Comments:

1. The authors should more explicitly discuss the broader implications of their findings. Are nanobodies being proposed as direct antimicrobial agents targeting RSH enzymes? The authors expressed the nanobodies in E. coli to test its effect. In therapeutics, how are they planning to introduce the nanobodies to the pathogens? Or alternatively, are they intended as models to guide rational drug design? This distinction is critical and should be clearly addressed in the discussion section.

2. The final figure is conceptually important but lacks sufficient detail to convey the regulatory landscape of RSH enzymes. To strengthen the impact of the work, the figure should be redrawn to include known regulators—such as DarB—and their binding sites, alongside the NB898 interactions; similarly Rsd and NB585 side-by-side. This would help readers understand how nanobodies mimic or interfere with natural effectors and clarify the rationale behind comparisons made in the text.

3. Given that nanobodies are central to the study, the introduction should provide a more thorough overview of their origin, production, and relevance to protein research. Currently, this background is deferred to the discussion (line 398 onward), which creates an abrupt transition and may hinder reader comprehension. Integrating this information earlier would better contextualize the study and improve narrative flow.

Minor Feedback (mostly typos)
You introduce APCPP as a nonhydrolyzable ATP analog at line 287 when APCPP was introduced in line 270. Consider defining APCPP, as you have done at line 287, earlier.
Line 88 "Camelid nanobodies are well stablished structural biology…" should be "established structural" biology.
Line 104-105 "Only the structure of the monofunctional hydrolase… which a revealed a compact" needs to remove the unnecessary "a". Rewrite to "which revealed a compact".
Line 117 "controlling the amino acid synthesis" can be rewritten for clarity. "controlling amino acid synthesis".
Line 117 "is leveraged in so-called" should be rewritten to "is leveraged in the so-called"
Line 124 "None of nanobodies" should be rewritten to "None of the nanobodies"
Line 136-139 The sentence beginning with "We reason that our RelA…" should be rewritten for grammatical correctness. "We reason that our RelA-modulating nanobodies Nb94 and Nb96 could have an impact on the virulence of E. coli, mimicking the effects of catalytically impaired genetic knockouts of RelA
Line 162 the word "Nb94" doesn't seem to connect to the rest of the sentence. Consider removing it.
Line 190 "to with synthetase", rewrite to "while the synthetase domain is completely occluded"
Line 236 "we probed the epitope define by a13" rewrite to "we probed the epitope defined by a13".
Line 272 "The structure shows the binding of both nucleotides" rewrite to "The structure shows that the binding of both nucleotides, indeed, promotes the open conformation.
Line 273 Add a linking word or phrase to the sentence beginning "APCPP" As it stands right now there is no transition from the last piece of information to this one.
Line 306 "SYNTH activity:" should be changed to "SYNTH activity,".
Line 365 Rsd is introduced here for the first time. It would make sense to introduce it along with the other interacting proteins

first mentioned in the introduction in lines 82-83
Line 379 “Thus, selective inactivation the TGS” rewrite to “Thus, selective inactivation of the TGS.”

Reviewer #3

(Remarks to the Author)
The manuscript by Nerom and coauthors reports a study focused on designing nanobodies that target bacterial proteins RSHs responsible for biosynthesis and hydrolysis of the important cellular alarmones (p)ppGpp. The authors showed that nanobodies can trap the proteins in particular conformations, which are relevant to the functional stages of RSHs. The study combines a multitude of methodological approaches, from X-ray crystallography and biochemistry to bacterial infection models, to dissect the mechanism of RSH function by the nanobodies. This reviewer has enjoyed reading the manuscript and found it to be potentially interesting to the broad readership of Nat. Comm. I would support the publication of the revised version after addressing minor issues listed below.
The manuscript is written well, and the data and findings are clear for the most part. There is no need for additional experiments to support the authors’ conclusions, except for some repeats for statistical purposes (such as ITC), if not done already. It was great to see the coordinates and maps submitted with the manuscript, as this made the reviewer’s job easier. The structures are pretty good, although the nucleotide-bound structure may need some minor tweaks. Both structures require further justification for the chosen resolution limits. There are also minor suggestions to improve the manuscript, especially in the methodological section.
Text and figures:
1) Fig. 1c. Please repeat the ITC experiment under optimal conditions. In the experiment presented in the manuscript, all heat was generated after the first two additions of the titrant, precluding accurate determination of the binding affinity. Since the binding affinity is high, obtaining higher-quality data should not be a problem. Indeed, the value reported in the text (line 130) is 157 nM, while the ITC titration (Fig. 1c) gave 80 nM.
2) SFig. 1b,c. It is unclear why H432E mutation would disrupt the interface. Please elaborate and show potential clashes.
3) Fig. 2a,b and Table S1. The Kd values in the text do not match the values in the table. It would also be beneficial to have accurate, not rounded, values in the table.
4) Line 202-204 and Table S1. Contrary to the text, the table shows the value for only one nanobody, not two.
5) SFig. 3a. Please provide a more detailed legend to explain this panel. What do the colors mean? What does the bottom “heatmap” represent? The red dashed line highlights the data that suggests interactions, not proves them.
6) Line 222-227. The data in SFig. 3a does not clearly show the increase in the uptake in these regions. A better quality zoomed-in presentation would be beneficial. The colors are pale. Perhaps the color scale could be adjusted similarly to Fig. 5a.
7) Line 231-233. The data in SFig. 3a does not show a significant decrease in the uptake in the 65-78 and 304-326 regions.
8) Fig. 2e,f show “positive” change, which is relatively small. A similar panel for a “control” unchanged region is necessary to show, as SFig. 3a shows many changes spread over the length of the protein.
9) Line 236-239. The D283A mutation caused a less than 10% increase in Kd, while other mutations were more deleterious. It is probably not a good idea to join this mutation with other mutations in the statement.
10) Line 255-256. Please show the T58-K280 interaction in Fig. 3F. It would be easier to follow the structural description if hydrogen bonds were shown with dashed lines.
11) I urge the authors to consider moving the mutagenesis of the interface after the structure. It makes it easier to analyze the effects of the mutations with the structure already presented. It is interesting that the D283A mutation, disrupting a strong H-bond, does not weaken the binding.
12) Line 258. The ED catalytic motif has not been presented yet. Please show it and the ppGpp binding site in the figure.
13) Line 261-263. Does the sentence “all signature structural elements of the activation of catalysis by the SYNTH domain” refer to the previously published data? If yes, a reference is required here.
14) Line 274-275. What do R245 and R273 exactly do with the adenine base? “Coordinate” is rather vague. Do the other mentioned residues form interactions with the triphosphate moiety? Fig. 4b would benefit from H-bonds and ionic interactions shown by dashed lines.
15) SFig. 3d,e. What are these unbiased maps? Are they the omit maps calculated before adding the ligands to the structure? What are the sigma levels of the maps? This is important to know as lines 276-277 mention low occupancy of GDP. The 2Fo-Fc maps for GDP are very poor: the nucleobase does not have a good density map even at 0.5 sigma level in the best case. I do not know how the authors generated the map shown in the figure. Please show a simulated annealing composite omit map at 1.0 level for the best molecule. In two molecules of the asymmetric unit, GDP is correctly modeled as pyrophosphate, and this could also be shown.
16) SFig. 4b,c. Please mention in the legend to this figure and other legends that these are AlphaFold models, not real structures.
17) Fig. 5f. Please show R215 and D31 in this figure.
18) Fig. 6 is confusing. Please keep the same colors for identical domains and use the same color for antibodies. Please label domains. Please specify whether these presentations are structures, models, or both.
19) Line 568. Khaki?
20) Please prepare Table S2 according to the standards of the field and the Nature journals template. Some information should either be described in Methods or omitted. However, some critical information, for example, groupings of atoms, needs to be clarified. “Solvent” atoms are actually water molecules, while “other” atoms are apparently metals and nucleotides. It would be beneficial to have nucleotides and metals presented in separate categories.
21) Table S2: Data collection and refinement statistics are not separated.
22) Table S2. What value of C1/2 was used to determine the resolution cutoff? It is usually 0.5 (or 0.3 in some cases) for the highest resolution shell. The table lists 0.122 for the Nb8908 structure. Please see structural comments below. Please show

the simulated annealing composite omit map for several key regions of the protein to demonstrate the quality of the map, which should match the expected resolution.
23) Line 705. Isothermal titration calorimetry
24) Line 705-716. Please provide more details about ITC parameters and the experiments involving antibodies. How many times was each titration performed? There is no SD or SE in Supplementary Table 1.
25) Line 718-738. Please provide a more detailed crystallization procedure here, including crystallization conditions. Were the structures obtained from the screening plates? If no, were screening plate conditions adjusted?
26) Line 743. What is a composition of a “suitable” solution?
27) How did the authors distinguish between Mg^{2+} and Mn^{2+} cations in the structures? Was it based on the anomalous map? The anomalous map for Mn1 is at the level of noise, and Mn5 does not have an anomalous map.
28) There are numerous typos, especially in Methods.
Structures:
Nucleotide-bound structure
29) The resolution cutoff of the structure needs further justification, as low CC1/2 of 0.411, along with poor completeness (53.5%) and subpar I/sI (1.1) in the highest resolution shell, suggests the overestimation of the resolution.
30) Mg5, Mg6, and Mg7 in the nucleotide-bound structure are likely waters, as they contact a positively charged side chain and are located at the hydrogen bond distance, not a coordination distance of this metal. Please make sure that all metals are assigned correctly.
31) What is the huge unassigned map by Asn152/C and Mn4? Is this another nucleotide molecule? It is present in all molecules of the asymmetric unit.
32) Some water molecules do not have 2Fo-Fc electron density map at 1.0 sigma level in both structures. What is the basis for modelling them?

Antibody-bound structure
33) Rmerge of 0.619 indicates that the structure was not processed in the correct space group, or the resolution was significantly overestimated, as could be the case with very low CC1/2. I do not recall any published structure with such a high Rmerge at this resolution. I believe the maximum I saw was around 0.25. However, the electron density map is quite good. The authors mentioned the high anisotropy of the crystals (4.99 Å in b)* that could be a factor impacting the merging statistics. Was anisotropy correction performed? The authors should revisit their merging statistics, clarify this issue, and provide additional details on merging and choosing the resolution cutoff.
34) The map is good, and there are no major issues with the structural model. In a few places, where the side chains were removed, I would leave some extra atoms, but this would not have an impact on the structure.

Version 1:

Reviewer comments:

Reviewer #1

(Remarks to the Author)
Despite some minor flaws in the initial version, I had been enthusiastic about the manuscript in the first place (and obviously still am; keep up the work on the subject...). My remarks have been sufficiently addressed by the authors' improvements on the manuscript. That being said, I can now recommend publication.

Reviewer #2

(Remarks to the Author)
The manuscript is greatly improved in its clarity. I only have a few additional minor suggestions:

Line 93: “stablished” should be changed to “established”.
Lines 66-68: Missing Hogg et al 2004 (PMID: 15066282) which first showed reciprocal regulation of Syn and Hyd domains in long RSH
Lines 114-117: Missing Pausch et al 2020 (PMID: 32937119) which is another structural study of off-ribosome Rel
Lines 323-324: Missing Mechold et al 2002 (PMID: 32937119) which is the early work that shows CTD truncations in bifunctional long RSH increases Syn activity

Reviewer #3

(Remarks to the Author)
The revised manuscript by Nerom et al. has been substantially improved. However, there are still a few overlooks that need to be addressed.
(1) All figures with ITC and Supplementary Table S1. What are Kds and errors (SD, SE) in each representative experiment in the figures? What are Kds and errors (SD, SE?) in each line of the table? How many times was each experiment repeated? This question has already been asked (point 24), but the authors failed to incorporate their answer into the

manuscript.
(2) Fig. 2c,d. The statistics of the experiments are not defined in the figure legend. What are the values? Mean with SD?
(3) Point 30. Mg12/E is unlikely to be a Mg2+ cation as it contacts positively charged side chains of Arg and Lys. It is either an anion or a water molecule.
(4) Point 32. The authors' answer is confusing. I recommend incorporating the cutoff criteria for automatic picking of water molecules, as some waters are unlikely to be picked up by, for example, Phenix's default parameters. However, Buster apparently picked them up. For example, HOH29/G has literally no density. Although water occupancy may vary, the density must be well above the noise. Several water molecules have a density at sigma level 0.5, basically the noise level.
(5) Point 22. SFig. 4. In the legend, please specify which density map is presented in panels a-c. In panels g, h, and I, are these maps simulated annealing composite omit maps as in the response to point 22? "Unbiased omit maps" is not a sufficient description.
(6) Fig. 4c, d. Please specify the type of map contour level in the legend.
(7) Fig. 4 legend. ITC is panel e, not d.
(8) Fig. 5a. Please indicate by a red dashed line box the region with increased deuterium uptake (K47-R48, etc.).
(9) Fig. 5b-d. A graph for "a control" peptide, without much uptake, such as residue 130-137, must be shown here.
(10) Fig. 5a. The R.F.U. scale for the top two images is not shown here.
(11) I think Figs. 1rev, 2rev, and Fig. 4rev could be incorporated into the supplementary Information section.
(12) Point 18. Fig. 6. Most colors in panel a look darker than in panels b and c, especially red, pink, and yellow. Please label domains. It is difficult to go back to Fig. 1a for color-coding. To avoid confusion, perhaps antibodies should not be shaded here, as they are not shown in the cartoon. Initially, I thought that they were presented in the structures, and many readers would assume the same.
(13) Point 20. Fig. S3. The need for atom-grouping is to show B-factors for each category, especially for metals and nucleotides, as they are not highly occupied. If occupancy is overestimated, the atoms will be "hot". This is still not done here despite the rebuttal letter saying that this issue was addressed. There are still "Other atoms" in B-factor section. A footnote should specify which ligands are included, as nucleotides are not the only ligands in the structures.

Open Access This Peer Review File is licensed under a Creative Commons Attribution 4.0 International License, which permits use, sharing, adaptation, distribution and reproduction in any medium or format, as long as you give appropriate credit to the original author(s) and the source, provide a link to the Creative Commons license, and indicate if changes were made.
In cases where reviewers are anonymous, credit should be given to 'Anonymous Referee' and the source.
The images or other third party material in this Peer Review File are included in the article's Creative Commons license, unless indicated otherwise in a credit line to the material. If material is not included in the article's Creative Commons license and your intended use is not permitted by statutory regulation or exceeds the permitted use, you will need to obtain permission directly from the copyright holder.
To view a copy of this license, visit https://creativecommons.org/licenses/by/4.0/

REPLY TO REVIEWERS

Reviewer #1 (Remarks to the Author):
In the current manuscript „Nanobody-mediated control of long RSH Rel and RelA catalysis by restriction of their conformational landscape“ by Van Nerom et al. (NCOMMS-25-68425) the labs of Vasili Hauryliuk and Abel Garcia-Pino build on their previous works about RSH enzymes and conceive nanobodies effective against the primary (p)ppGpp enzyme Rel/RelA, including a rigorous analysis of their modes of action. As to my knowledge, the presented nanobodies are the first molecules acting allosterically on Rel activity with nanomolar affinities, whereas the handful of small molecule inhibitors described in literature act competitively with micromolar affinities. The description of these first-in-class nanobodies against Rel thus makes me quite enthusiastic about the paper.

In short, in the current study the authors raised nanobodies in camels that bind to the Escherichia coli RelA protein in vitro, impact E. coli growth upon overexpression in vivo and are also effective in an infection model. For further structural, biochemical and biophysical studies the authors switched to Rel from a different organism (Chlorobaculum tepidum, Ct) due to its better behaviour in vitro and provide mostly conclusive evidence on the modes of action of two distinct nanobodies raised against different states of the bifunctional RelCt protein. These results are put into context of ‚endogenous ’Rel activity modulators, i.e., factors that govern Rel activity in bacteria.

The manuscript as a whole is well-written and describes the structural biology of Rel (and how it is impacted differently by the employed nanobodies) well even for the non-expert reader. The analyses in the study employ a timely set of methodology (X-ray crystallography and occasionally Alphafold-modelling, affinity and activity assays, hydrogen/deuterium exchange mass spectrometry) that is mostly well-executed and described.

Thematically, I do not see major blank pages in the presented study at hand. I could have only wished for more in vivo experimentation and application of the nanobodies with techniques other than their overexpression in bacterial host such as E. coli (experiments in Fig. 1). This would adress the most obvious next step dealing with pharmacology of the drugs, primarily their delivery. However, I do understand that this would eventually require an entire additional study that the authors are currently anyways working on at probably.

I can nevertheless and unfortunately not recommend publication of the current study right away due to some shortcomings in methodology related to the AMPCPP/GDP-bound RelCtNTD structure by X-ray crystallography and the mode how Nb898 affects Rel activity by HDX-MS. Both of these do not put into question the key findings of the study, i.e. generation of nanobodies impacting Rel activity, and as soon as these issues are adressed I strongly recommend publication of this study.

We are grateful to the reviewer for their suggestions regarding the improvement and presentation of the X-ray structure of Rel_{Ct}^{NTD} bound to APCPP/GDP, as well as the HDX-MS data. We have now revised the structural model in accordance with the reviewer’s comments, see below. In the case of GDP, only the ribose 5′-pyrophosphate is now shown, together with an OMIT map contoured at 1σ in the main figures (Fig. 4d), while the pyrophosphate moieties observed in the three other chains are shown in Supplementary Fig. 3g-i.

Major points

1) Assessment of the crystal structures (mainly AMPCPP/GDP-bound RelCtNTD)

The space groups assigned in the ‚Full wwPDB X-ray Structure Validation Report ’are not coherent with the space groups mentioned in the ‚Supplementary Table S2. X-ray data collection and processing’.

We appreciate these constructive and helpful remarks and we now have corrected the table accordingly. We would like to reassure the reviewer that both space groups remain the same. In the table the SG is listed as $P2_1$ and in the validation report as P1211, but this, is fact, the same SG just the notation is different. In the revised version table S2 was updated to avoid possible misunderstandings.

Also, in this table the completeness in the high resolution shell of the AMPCPP/GDP-bound structure seems very low. The authors would please double-check these.

Regarding the completeness of the data set, as the reviewer noticed, the data is highly anisotropic. Therefore, as explained in the Methods section, we use the STARANISO software (https://staraniso.globalphasing.org) to address the anisotropy problem which resulted in a very good map. This analysis results in different resolution limits in each axis and overall loss of completeness; however the data quality improves significantly by reducing the anisotropy effect. The current resolution limits were not imposed by determined by the software based on the well-accepted guidelines by Karplus and Diederichs (Linking crystallographic model and data quality; Science, 2012 DOI: 10.1126/science.1218231). This approach has resulted in very good statistics and map for this type of data, and, therefore, we are confident in the current resolution cutoff and data.

The structure of the RelCtNTD : Nb898 complex seems fine within the scope of the limited resolution of 3.15 Angstrom. However, related to the RelCtNTD : GDP : APCPP complex structure I see a potential issue in the Validation Report and the provided electron density maps for the GDP ligand:

As per the validation report the GDP is modelled entirely into the density in chains A and B (p. 31+33). Generally, the 0.7 rmsd cut-off for the 2Fo-DFc is very liberal and a cut-off of 1-1.5 would be more conservative (eventually set arbitrarily by the generating software of the Validation Report?). But even at this low 0.7 cut-off the GDP is poorly explained by density, in particular the guanine base. In chains C and D (p. 32+33) only a pyrophosphate (instead of GDP) is placed in the Validation Report. The density of the APCPP (APC) in contrast looks convincing in all four chains (p. 33-36). Inspection of the mtz files suggests a rather poor agreement of experimental density and the GDP modelling - the density for GDP looks deceptively flat to me instead of harboring some bulbs here and there (substitutions at base, ribose OH's and phoshphate, compare to APC density). In particular the guanine base exhibits almost no density at all although one would expect well-defined density for this particular entity given ist mode of coordination (π-stacking with a tyrosine provided by the G-loop). The authors themselves mention a low occupancy of the GDP (l. 276f) and it may just be that the Validation Report does not properly reflect this – but I hope this isn't just HEPES (from the purification buffer) or a PEG fragment (from the crystallization conditions) bound to RelCtNTD : APCPP instead of GDP. An unbiased Fo-Fc map of GDP, which seems OK'ish to me by eye, is provided in SFig. 3e but without mention of neither chain ID nor the contour level. Please amend these.

As suggested, we have now revised the X-ray structure of Rel_{Ct}NTD bound to APCPP/GDP. In the amended coordinates only the ribose 5'-pyrophosphate of GDP is modelled. This is shown in SFig. 4d together with an OMIT map at 1σ. For the other chains the pyrophosphates modelled in the structure are now also shown in SF 4g-i. As suggested, the identity of each chain ID is now included in the figure legend.

→ I am convinced that GDP binds to APC-preloaded RelCtNTD (ITC data in Fig. 4f) and also admit that this SYNTH substrate-bound structure is not the major finding of the manuscript but nevertheless must urge the authors to check this particular dataset again thoroughly.

The dataset was thoroughly checked as advised (see above). The crystallisation condition does not involve HEPES (the buffer was 25 mM Tris). Given the similarity of the pose in the active site compared to other complexes of RSH enzymes with GDP, the most likely explanation is that this molecule is indeed GDP. As mentioned above, the structure was revised as advised, and coordinates now contain the ribose 5'-pyrophosphate of the GDP molecule in one of the chains with the others modelled as a pyrophosphate.

In conjunction with that the authors argue in ll. 292ff about the low GDP occupancy being in agreement with ITC results. I can't follow this argumentation given that APCPP and GDP were both present at 10 mM during crystallization (methods section), which should be absolutely sufficient given the ITC-determined two-digit micromolar Kd's.

We fully appreciate the point made by the reviewer, and this line has been removed in the revised version.

2) Assessment of the HDX-MS datasets (mainly Nb898 binding to RelCt)

Unfortunately I could not assess the HDX-MS data in detail despite having received the reviewer login token to the PRIDE database entry. Reason is that I could not open the DynamX file despite using the very same version number (3.0) mentioned in the data analysis procedure of the entry. Opening the DynamX sessions stopped when reading the lock mass data; I expect the same problem would occur after publication of the dataset.

The authors should check this PRIDE entry itself for some inconsistencies and amend (e.g., it states CapRelSJ46 and Gp57 as search sequences, which appears to be a copy-paste error from a previous submission on TA systems; a FASTA and the PLGS output may also be provided).
The manuscript is currently lacking a HDX source data file (or similar) stating HDX-MS experiment's characteristics and HDX values (compare to the tables provided in the recommendations by Masson GR et al., Nat Methods 2019), which is critical for non-expert readers to assess the data. Such a dataset could have ruled out my concerns related tot he mode of action how Nb898 affects Rel activity:

We appreciate the concerns regarding the Nb898 - Rel HDX dataset. We believe the proposed mode of action of Nb898 is strongly supported beyond the HDX data, by the crystal structure in complex with the enzyme and a large set of point mutants probing by ITC the interface. Therefore, we wanted to clarify that in our opinion the binding epitope is not questioned here. Nevertheless, we acknowledge the issues raised by the reviewer which have been addressed in the revised version.

1) The Pride entry has been amended according to the recommendations. The FASTA file with the sequences used for the analysis and the PLGS outputs and the HDX reporting table have been added. The Pride ID is the same, PXD067451.

2) The HDX reporting table is now also in the supplementary info (Supplementary Table 2).

3) The uptake plots are available on the public data repository figshare with the following credentials. "Martens, Chloe (2025). uptake plots RelCT-NTD with nanobodies 585 and 898. figshare. Dataset. https://doi.org/10.6084/m9.figshare.30264736.v1".

SFig. 3 highlights a pronounced HDX change of Rel in presence of Nb898 at res. approx. 280, which is consistent with the binding site by the presented crystal structure. Then, two peptides are presented (Figs. 2e and f), which shall provide evidence for secondary effects of Nb898 besides the binding event.

- A) The differences exhibited by the 118-127 and 209-221 peptides are of very small magnitude, and if these fluctuations are considered significant, what is with other areas such as residues 20-35, 155-170, 240-250?
- B) I do not consider the changes exhibited in the peptides 118-127 and 209-221 as confident due to their dynamics. In 118-127 the behaviour is odd in that HDX gets lower with increasing deuteration time suggesting a high level of experimental fluctuation for this seemingly disordered (by HDX progression over time) region. In 209-221 the red curve with Nb898 is just shifted to higher uptake values by a small margin, which can easily come from divering peptide ion intensities or inconsistent assignments of undeuterated ion clusters.

We thank the reviewer for his critical assessment of the data. The significance of the difference in exchange at these two regions was initially considered meaningful based on the butterfly plot provided by DynamX (**Fig. 1rev**), where a ±2.33σ threshold—corresponding to a 98% confidence interval—highlighted these two regions (116-127 and 209-221) as significantly different, as well as the other regions pointed out by the reviewer (20-31, 147-169 and 239-253).

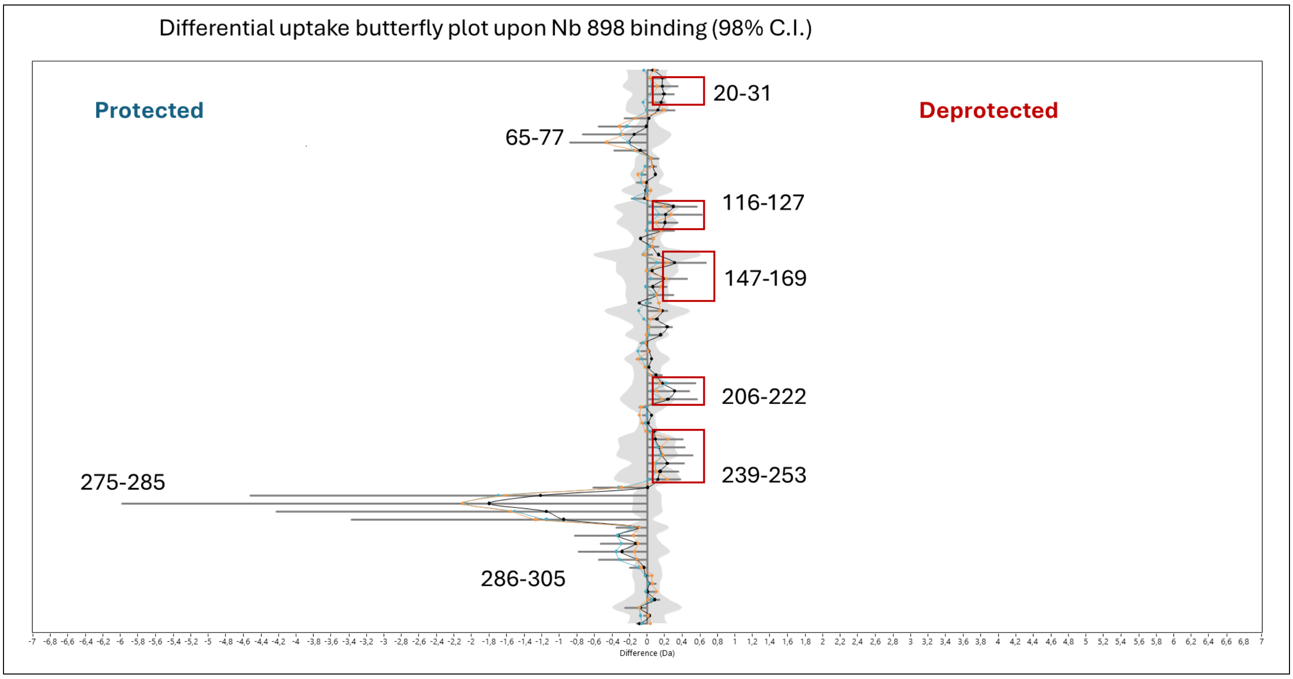

Fig. 1rev. Bar plot representing differences in deuterium uptake between free- and Nb898 bound Rel, with each bar corresponding to a unique peptide from the peptide library (ordered from N- to C- terminus based on the first amino acid of each peptide). The X-axis represents the total difference in D uptake for a given peptide, calculated as the sum of the mass differences between free and bound at each time point. Positive bars indicate protection of the corresponding peptide upon binding. Deuterium uptake differences are shown for each peptide and labeling time point: 0.5 min (orange), 5 min (blue), and 60 min (black). Light grey shades show the differential uptake s.d for each peptide.

However, the validity of the confidence intervals provided by DynamX has been called into question, including for the reasons mentioned by the reviewer and because the way the SD is calculated is not publicly available to my knowledge (grey shading **Fig.1 rev**). Based on the suggestion of the reviewer, we applied a more stringent and commonly accepted analysis of the differential uptake data using the Deuteros 2.0 software (https://academic.oup.com/bioinformatics/article/37/2/270/5877424), which evaluates the

significance of ΔHDX both at the peptide level and at the dataset level, following recommendations

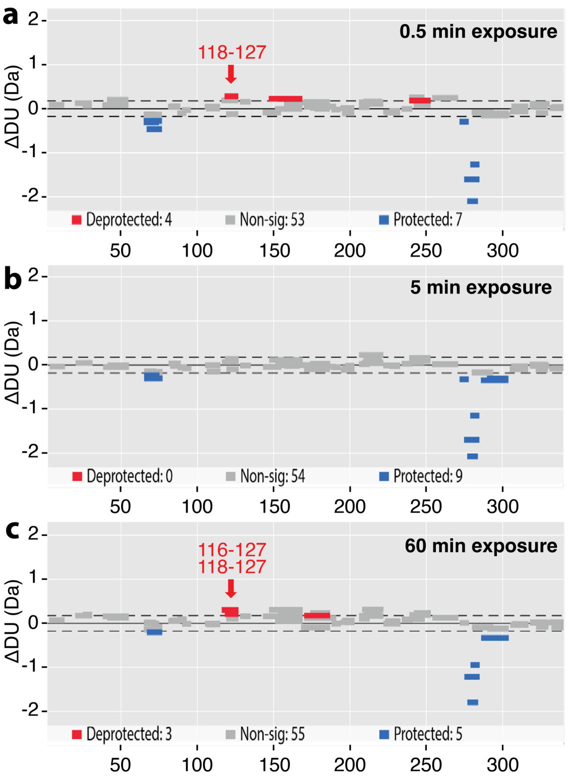

Fig. 2rev. Differential uptake Woods plot upon Nb 898 binding (98% C.I., pvalue > 0.02). Comparison of the relative deuterium uptake of Rel in the presence and absence of Nb898, after incubation at room temperature in deuterated solvent for 30seconds, 5, or 60 minutes. The dotted lines represent the 98% confidence interval, which indicates the level of difference of deuterium uptake between the two compared states is statistically significant. Bars represent individual peptides. Bar length corresponds to peptide size. Red coloured bars indicate statistically significant deprotected peptides, (region 118-127 is highlighted).

from the HDX-MS community (https://pubs.acs.org/doi/full/10.1021/acs.analchem.9b01325). According to this analysis (**Fig. 2rev**), most of the regions that appeared deprotected in the butterfly plot from DynamX do not display statistically significant differences. Peptides from region 116–127 are considered to show a significant difference at two of the three timepoints when using a 98% confidence interval. Nevertheless, the reviewer rightly pointed out that the behaviour of this peptide cluster is unusual. This is observed only in this region and for the three peptides covering it, which indeed complicates the interpretation of the data in terms of structural changes.
Based on this analysis, and to distance ourselves from over interpreting our data, we have now removed the following paragraph from the main text:

"The overall effect of Nb898 binding was an increased deuterium uptake (ΔHDX) involving both catalytic regions that radiates from the central region of the enzyme (Fig. 2e-f and Supplementary Fig. 3a). The highest uptake increase localizes to the allosteric hub involving the α6α7 active site motif (residues 101 - 135) and α11 (residues 201 - 236) that mediate the coupling between SYNTH and HD domains18,20. The higher degree of uptake observed in this crucial allosteric region (Fig. 2e-f) suggests that the binding of Nb898 triggers a more dynamic state of the enzyme in which the SYNTH active site is more exposed and primed for substrate binding and catalysis. This aligns closely with prior structural evidence showing that α11 unwinds in Rel and RelA upon activation of the SYNTH domain18,20."

→ During preparation of the HDX-MS data in above-mentioned format they could check the two areas in question again for, e.g., more redundant peptides, to substantiate the claims on Nb898 action on Rel activity.

See previous paragraph.

Minor points
• Some typos, e.g.:
- l. 333: Sentence/argument is not finished

Corrected.

- l. 339: missing an ATP „binding“

Corrected.

- l. 756: space missing between 20% and glycerol

Corrected.

- SI table 3: „subsituted“ throughout

Corrected.

→ These are just the one's I spotted when reading. The authors will certainly find some more and amend these.

We thank all the reviewers for pointing out these typos in the manuscript. The revised manuscript has now been thoroughly proofread.

• I am missing a (short) description of cloning methodology (incl. a supplementary table with oligos)

As requested, a list of the cloning methodology for the nanobodies was included in the Methods section (this includes a list of the oligonucleotides which is now part of Supplementary Table S4. All the RSH constructs of recombinant WT enzymes and mutants (from this work) were generated by GenScript (https://www.genscript.com). This is also mentioned in the Methods section.

• Can the authors comment in the manuscript on the endergonic ITC curve of GDP into Nb898-bound RelCt in Fig. 4g? This is just unexpected (compare to GDP into APCPP-bound RelCt in Fig. 4f) and could indicate more action going on in the area of the G-loop, in which's close vicinity Nb898 is also implicated to bind.

As suggested by the reviewer, we have added a paragraph in this section to provide an analysis of the thermodynamics of GDP binding.

*"Interestingly, the binding of GDP to Rel in the presence of APCPP is enthalpy driven, whereas Nb898 induces a clear switch to an entropically driven interaction (**Supplementary Table S1**). This reflects the allosteric exposure of hydrophobic patches within the large SYNTH active site (including the G-loop), which are associated with highly ordered water molecules. Upon GDP binding to the Rel_{Ct}-Nb898 complex, release of these waters into bulk solvent likely generates a substantial entropic gain that dominates the binding free energy. Moreover, Nb898 likely pre-organizes Rel into a more restricted, bound-like conformational ensemble, thereby minimizing the conformational entropy penalty associated with complex formation. Such changes in binding energetics are consistent with enthalpy-entropy compensation commonly observed upon perturbation of biomolecular recognition."*

• ITC methodology (ll. 705ff):

- l. 709 → I never saw nucleotides being dissolved at such high concentrations. Are you sure or was it actually micromolar?l. 713 → I assume these were the final concentrations of the prep. Concentration of Rel in the cell should be stated as well. Another column in Supp. Table 1 would be a good fit unleast you don't want to mention this in each ITC-depicting figure caption.

Indeed, apologies for the typo, the concentrations mentioned in this part are, indeed, in micromoles.

• Crystallization (ll. 718ff):

- Would be nice if authors could explicit the crystallization conditions in which crystal were obtained instead of stating the well of the screen alone.

Changed in the Methods section as requested.

• The separately-supplied preparation of the camelid nanobodies should be integrated into the materials & methods section.

Changed in the Methods section as requested.

We have shortened this part by moving to the Methods section all the details involving the preparation of the nanobodies:

"Generation of RSH-targeting nanobodies

Following established protocols58, peripheral blood lymphocytes were collected from the immunized llamas, and RNA was extracted for cDNA synthesis. After the first amplication with primers call001 and call002, the variable domains of heavy-chain-only antibodies were amplified using primers 229 and 230 and cloned into a Golden Gate variant of the pMESy4 phage display vector (KF415192) using SapI to construct camelid nanobody libraries. After two rounds of selection against either RelAEc or RelCt – either in unbound state or in the presence of GDP or APCPP – we successfully isolated after phage display selection58 a set of different candidate binders that were classified according to the sequences of the third complementarity determining region (CDR3, see Supplementary Fig. 1a)."

Reviewer #2 (Remarks to the Author):

It is well established that Rel/SpoT homolog (RSH) enzymes, the central regulators of the bacterial stringent response, adopt distinct conformational states to carry out either their synthetase or hydrolase functions. In this study, Nerom and colleagues introduce a novel set of camelid-derived nanobodies capable of locking long RSH enzymes into these discrete functional states. By immunizing llamas with RSH proteins, the authors generated nanobodies that selectively stabilize alternative conformations, effectively enabling constitutive activation or repression of enzymatic activity—independent of ribosomal regulation.

Through an integrated approach combining structural prediction, high-resolution structural analysis, biochemical assays, and microbiological techniques, the authors mapped the nanobody binding sites and elucidated their mechanisms of action. Notably, they demonstrated that overexpression of these nanobodies in E. coli reduced bacterial virulence in a Galleria mellonella (wax moth) infection model, highlighting the potential of nanobodies as tools for modulating bacterial pathogenicity and informing antimicrobial strategies.

This work presents an interesting case for the use of nanobodies in RSH research, both as molecular probes to dissect regulatory mechanisms and as potential scaffolds for therapeutic development.

Major Comments:

1. The authors should more explicitly discuss the broader implications of their findings. Are nanobodies being proposed as direct antimicrobial agents targeting RSH enzymes? The authors expressed the nanobodies in E. coli to test its effect. In therapeutics, how are they planning to introduce the nanobodies to the pathogens? Or alternatively, are they intended as models to guide rational drug design? This distinction is critical and should be clearly addressed in the discussion section.

We are grateful for the interest of the reviewer; this is an early-stage exploratory study aimed at probing the role of RSH enzymes in growth and virulence beyond genetic approaches. While the nanobodies used are not therapeutics and cannot be directly discussed as antimicrobials, their high affinity, specificity, and on-target effects provide strong validation of RSH enzymes as druggable targets. Given the challenges of intracellular delivery of folded proteins, our goal is to use these nanobodies as molecular tools to guide rational drug design. For example, they offer valuable starting points for pharmacophore development and reveal new allosteric hotspots amenable to

small-molecule targeting. As the reviewer suggested, this distinction has now been clarified in the revised discussion section:

"It is important to emphasize that the nanobodies developed in this study are not intended as therapeutic agents, but rather as exploratory tools to dissect the conformational regulation of long RSH enzymes and the stringent response. While these nanobodies robustly modulate long RSH activity in vitro and in the Galleria infection model, their value lies in establishing long RSH enzymes as bonafide targets to develop novel antibacterials. By revealing discrete allosteric hotspots and defining conformational states that correlate with catalytic output, these nanobodies offer a structural and conceptual framework for the rational design and development of small-molecule modulators targeting the stringent response and inform future studies aimed at exploiting the enzymatic output of long RSHs."

2. The final figure is conceptually important but lacks sufficient detail to convey the regulatory landscape of RSH enzymes. To strengthen the impact of the work, the figure should be redrawn to include known regulators—such as DarB—and their binding sites, alongside the NB898 interactions; similarly Rsd and NB585 side-by-side. This would help readers understand how nanobodies mimic or interfere with natural effectors and clarify the rationale behind comparisons made in the text.

This is a valid and complex point. We agree that a direct side-by-side comparison could further illustrate how nanobodies mimic natural effectors. However, at present, such an analysis is only feasible for the case of DarB and Nb898 (being the only pair of naturally occurring modulators and Nb with well-defined binding sites). To clarify, the comparisons made in our study are based on the functional outcomes of these endogenous modulators (such as DarB) on the long RSH enzymes. The precise binding sites of Rsd, ACP, or NirD remain undefined beyond the general domain-level interactions. This is further complicated by the fact that Rsd, for instance, is not present in *A. baumannii*. Therefore, any additional structural comparison would be speculative. This rationale has now been explicitly stated in the figure legend.

Nevertheless, we agree that such contextualization enhances the overall message of the paper. For this reason, the schematic comparison in Supplementary Fig. 5b-c is being added as a clear reference alongside Fig. 6 in the revised manuscript. This part now reads:

"Analogously, Nb898 binds the structural epitope that we have earlier shown in the case of B. subtilis Rel to be recognized by SYNTH-stimulatory factor DarB53. Neither Nb898 nor DarB make direct contacts with the active site of the enzyme to enhance (p)ppGpp synthesis. Rather, both modulators counter the formation of the hydrolase-compatible τ-state by preventing the interaction between the RRM domain and the SYNTH domain Supplementary Fig. 4e-f."

3. Given that nanobodies are central to the study, the introduction should provide a more thorough overview of their origin, production, and relevance to protein research. Currently, this background is deferred to the discussion (line 398 onward), which creates an abrupt transition and may hinder reader comprehension. Integrating this information earlier would better contextualize the study and improve narrative flow.
As suggested by the reviewer in the revised manuscript we now provide a more detail overview of the use of nanobodies and their direct applications to structural biology:

"Camelid nanobodies are well stablished structural biology tools used to study the conformational landscape of proteins38-40. Their unique ability to recognize and stabilize specific conformational states of proteins aided by the unique complementarity determining regions (CDRs), has enabled unprecedented insights into dynamic and transient molecular mechanisms, that are otherwise challenging to capture41-44. By selectively modulating allosteric sites or locking proteins in functionally relevant states, nanobodies have illuminated fundamental principles of protein folding, signaling, and catalysis across a wide range of biological systems41-44."

Minor Feedback (mostly typos)
You introduce APCPP as a nonhydrolyzable ATP analog at line 287 when APCPP was introduced in line 270. Consider defining APCPP, as you have done at line 287, earlier.

As suggested, introduced earlier in line 119, section: "Generation of high affinity nanobodies against RSH enzymes" (the first section of the paper).

Line 88 "Camelid nanobodies are well stablished structural biology…" should be "established structural" biology.
Changed as suggested.

Line 104-105 "Only the structure of the monofunctional hydrolase… which a revealed a compact" needs to remove the unnecessary "a". Rewrite to "which revealed a compact".
Changed as suggested.

Line 117 "controlling the amino acid synthesis" can be rewritten for clarity. "controlling amino acid synthesis".
Changed as suggested.

Line 117 "is leveraged in so-called" should be rewritten to "is leveraged in the so-called"
Changed as suggested.

Line 124 "None of nanobodies" should be rewritten to "None of the nanobodies"
Changed as suggested.

Line 136-139 The sentence beginning with "We reason that our RelA…" should be rewritten for grammatical correctness. "We reason that our RelA-modulating nanobodies Nb94 and Nb96 could have an impact on the virulence of E. coli, mimicking the effects of catalytically impaired genetic knockouts of RelA
Changed as suggested.

Line 162 the word "Nb94" doesn't seem to connect to the rest of the sentence. Consider removing it.
Changed as suggested.

Line 190 "to with synthetase", rewrite to "while the synthetase domain is completely occluded"
Changed as suggested, now reads:
"*These states involve a compact HDON-compatible τ-state, in which both catalytic domains are close together with synthetase active site completely occluded.*"

Line 236 "we probed the epitope define by a13" rewrite to "we probed the epitope defined by a13".
Changed as suggested.

Line 272 "The structure shows the binding of both nucleotides" rewrite to "The structure shows that the binding of both nucleotides, indeed, promotes the open conformation.
Changed as suggested.

Line 273 Add a linking word or phrase to the sentence beginning "APCPP" As it stands right now there is no transition from the last piece of information to this one.
Changed as suggested.

Line 306 "SYNTH activity:" should be changed to "SYNTH activity,".

Changed as suggested.

Line 365 Rsd is introduced here for the first time. It would make sense to introduce it along with the other interacting proteins first mentioned in the introduction in lines 82-83
Changed as suggested.

Line 379 “Thus, selective inactivation the TGS” rewrite to “Thus, selective inactivation of the TGS.”
Changed as suggested.

Reviewer #3 (Remarks to the Author):
The manuscript by Nerom and coauthors reports a study focused on designing nanobodies that target bacterial proteins RSHs responsible for biosynthesis and hydrolysis of the important cellular alarmones (p)ppGpp. The authors showed that nanobodies can trap the proteins in particular conformations, which are relevant to the functional stages of RSHs. The study combines a multitude of methodological approaches, from X-ray crystallography and biochemistry to bacterial infection models, to dissect the mechanism of RSH function by the nanobodies. This reviewer has enjoyed reading the manuscript and found it to be potentially interesting to the broad readership of Nat. Comm. I would support the publication of the revised version after addressing minor issues listed below.
The manuscript is written well, and the data and findings are clear for the most part. There is no need for additional experiments to support the authors ’conclusions, except for some repeats for statistical purposes (such as ITC), if not done already. It was great to see the coordinates and maps submitted with the manuscript, as this made the reviewer’s job easier. The structures are pretty good, although the nucleotide-bound structure may need some minor tweaks. Both structures require further justification for the chosen resolution limits. There are also minor suggestions to improve the manuscript, especially in the methodological section.
Text and figures:

1) Fig. 1c. Please repeat the ITC experiment under optimal conditions. In the experiment presented in the manuscript, all heat was generated after the first two additions of the titrant, precluding accurate determination of the binding affinity. Since the binding affinity is high, obtaining higher-quality data should not be a problem.

We are grateful to the reviewer for pointing this out. We replaced the panel shown in 1c by a repetition of this titration (now Fig. 1e).

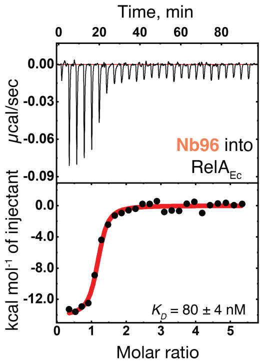

Fig. 3rev. Binding of Nb96 into *E. coli* RelA monitored by Isothermal Titration Calorimetry (this titration is now shown in Fig.1e, main text).

2) Indeed, the value reported in the text (line 130) is 157 nM, while the ITC titration (Fig. 1c) gave 80 nM.
We apologize for the typo, the revised version has been amended with the correct value of 80nM (reported in figure in the previous version). This was also fixed in Supplementary Table S1.

3) SFig. 1b,c. It is unclear why H432E mutation would disrupt the interface. Please elaborate and show potential clashes.

In the case of the interface with Nb94, H432 is found in a hydrophobic pocket and close to D99 and D106. The introduction of a negative charge from the H432E substitution into this pocket and close to the other two negative charge likely compromises the integrity of the interface leading to the drop in affinity. In the case of Nb96 (as illustrated in the figure), given the proximity of H432 to D55 of Nb96 and D434 of RelA, the substitution of the favorable His side chain by a negatively charge glutamate residue is likely the element that compromises the interface leading to the drop in affinity. In the revised version of the figure, we have included in panel b the binding pocket for H432 and as requested by the reviewer we explain the source of clashes in the main text:

"In both cases, the introduction of a negative charge via the H432E substitution likely destabilizes the binding interface, resulting in reduced affinity. For the interaction with Nb94, H432 is buried within a hydrophobic pocket and further coordinated by D99 and D106, while in the case of Nb96, H432 lies in proximity to D55 and RelA's D434 (Supplementary Fig. 1b-c)."

3) Fig. 2a,b and Table S1. The Kd values in the text do not match the values in the table. It would also be beneficial to have accurate, not rounded, values in the table.
Modified as requested by the reviewer.

4) Line 202-204 and Table S1. Contrary to the text, the table shows the value for only one nanobody, not two.
Table S1 now shows the binding parameters for the interaction of Nb898 into RelCt and Nb585 into RelCt.

5) SFig. 3a. Please provide a more detailed legend to explain this panel. What do the colors mean? What does the bottom "heatmap" represent? The red dashed line highlights the data that suggests interactions, not proves them.
The missing information has now been added to the figure legend and figure panel.

6) Line 222-227. The data in SFig. 3a does not clearly show the increase in the uptake in these regions. A better quality zoomed-in presentation would be beneficial. The colors are pale. Perhaps the color scale could be adjusted similarly to Fig. 5a.

See Reviewer 2. Based on the suggestions and comments of the reviewers we have now reinterpreted the data and rewritten our conclusions regarding the effect of Nb898 on Rel.

7) Line 231-233. The data in SFig. 3a does not show a significant decrease in the uptake in the 65-78 and 304-326 regions.

We performed a more refined analysis of the statistical significance of the differential data (see our response to Reviewer 1 above) and confirmed that the differential uptake (protection) in the 65–77 region is statistically significant (**Fig. 4rev**, Woods plot shown below). In contrast, the moderate protection observed in the 304–326 region is not statistically significant. This has now been removed from the main text.

8) Fig. 2e,f show "positive" change, which is relatively small. A similar panel for a "control" unchanged region is necessary to show, as SFig. 3a shows many changes spread over the length of the protein.

See Reviewer 2. Based on the suggestions and comments of the reviewers we have now reinterpreted the data and rewritten our conclusions regarding the effect of Nb898 on Rel.

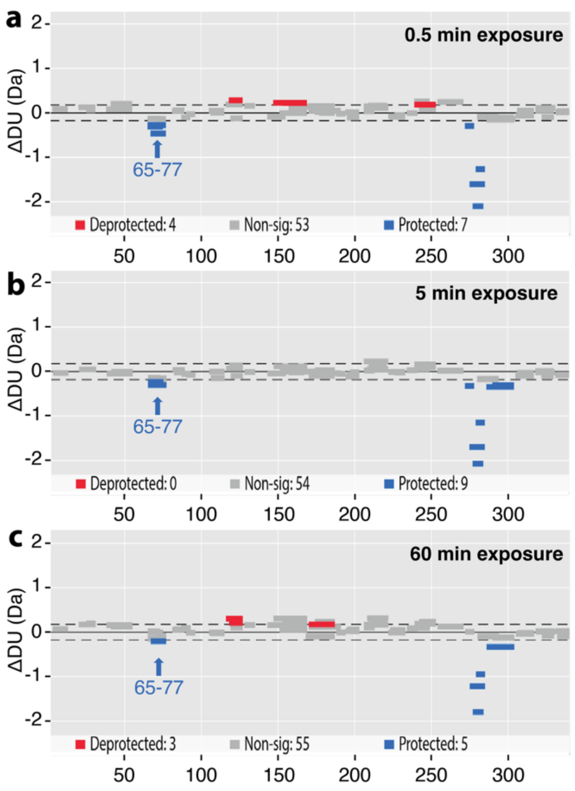

Fig. 4rev. Differential uptake Woods plot upon Nb 898 binding (98% C.I., pvalue > 0.02). Comparison of the relative deuterium uptake of Rel in the presence and absence of Nb898, after incubation at room temperature in deuterated solvent for 30seconds, 5, or 60 minutes. The dotted lines represent the 98% confidence interval, which indicates the level of difference of deuterium uptake between the two compared states is statistically significant. Bars represent individual peptides. Bar length corresponds to peptide size. Blue colored bars indicate statistically significant deprotected peptides. Region 65-77 showing statistically significant protection is labeled.

9) Line 236-239. The D283A mutation caused a less than 10% increase in Kd, while other mutations were more deleterious. It is probably not a good idea to join this mutation with other mutations in the statement.

Changed as requested:
"In all cases except D283A (which resulted in only a minor change in the Kd) we detected an increase in the binding Kd of the RelCt variants compared to the WT RelCt, confirming the binding epitope."

10) Line 255-256. Please show the T58-K280 interaction in Fig. 3F. It would be easier to follow the structural description if hydrogen bonds were shown with dashed lines.

There seems to be a slight confusion here. T59 hydrogen-bonds D283. This HB is now shown in the figure.

11) I urge the authors to consider moving the mutagenesis of the interface after the structure. It makes it easier to analyze the effects of the mutations with the structure already presented. It is interesting that the D283A mutation, disrupting a strong H-bond, does not weaken the binding.

This HB (T59-D283) is the only real HB from the interface, which is mostly hydrophobic, as shown in the figure. Therefore, it is not surprising that the D283A substitution has only a minor effect. Regarding rearranging the mutagenesis work. These residues were selected based on the observations from the HDX-MS data, and it pairs well with this section of the paper, so we prefer to leave it as it is.

12) Line 258. The ED catalytic motif has not been presented yet. Please show it and the ppGpp binding site in the figure.

The requested panel is now included as Fig. 3g.

13) Line 261-263. Does the sentence "all signature structural elements of the activation of catalysis by the SYNTH domain" refer to the previously published data? If yes, a reference is required here.

Reference 19 (Tamman et al., Nat Chem Biology. 2020) has been inserted here.

14) Line 274-275. What do R245 and R273 exactly do with the adenine base? “Coordinate” is rather vague. Do the other mentioned residues form interactions with the triphosphate moiety? Fig. 4b would benefit from H-bonds and ionic interactions shown by dashed lines.

Thanks for the remark, the nature of these interactions has been clarified in the revised text and the suggested modifications have been introduced in Fig. 4b.

"The adenine base is strongly coordinated via π-π interactions by R245 and R273 whereas the triphosphate moiety is held in position by interacting with R245, K247, S251 and K255 (Fig. 4b). Unfortunately, GDP is bound with lower occupancy and only the 5′-pyrophosphate stabilized by residues K255, K261, and K303 could be unambiguously modelled."

15) SFig. 3d,e. What are these unbiased maps? Are they the omit maps calculated before adding the ligands to the structure? What are the sigma levels of the maps? This is important to know as lines 276-277 mention low occupancy of GDP. The 2Fo-Fc maps for GDP are very poor: the nucleobase does not have a good density map even at 0.5 sigma level in the best case. I do not know how the authors generated the map shown in the figure. Please show a simulated annealing composite omit map at 1.0 level for the best molecule. In two molecules of the asymmetric unit, GDP is correctly modeled as pyrophosphate, and this could also be shown.

As requested by the reviewer Figure 4c-d and SF4 g-i, show the a simulated annealing omit map at 1.0 level for APCPP and the modelled groups of GDP in each chain. This includes the ribose 5’-pyrophosphate from chain A, and the pyrophosphates of chains B-D.

16) SFig. 4b,c. Please mention in the legend to this figure and other legends that these are AlphaFold models, not real structures.

Modified as requested.

17) Fig. 5f. Please show R215 and D31 in this figure.
Modified as requested.

18) Fig. 6 is confusing. Please keep the same colors for identical domains and use the same color for antibodies. Please label domains. Please specify whether these presentations are structures, models, or both.

We appreciate the reviewer’s comment. To improve clarity and consistency, we have added a domain schematic of RSH enzymes in Fig. 1a, which introduces a standardized color-coding for the enzymatic domains of Rel, RelA, and SpoT. This color scheme is now applied consistently in all subsequent figures, including Fig. 6, unless otherwise noted for clarity.
We would also like to clarify that the structures shown are cartoon representations, not experimentally derived models. This is now explicitly stated in the figure legend:

“Cartoon representation of observed conformational states of long-RSH enzymes (τ, relaxed, and elongated). These conformations are stabilized by cellular binding partners to modulate RSH activity. Enzymatic domains are colored according to the convention introduced in Fig. 1a.”
Additionally, the coloring of nanobody-related boxes has been made consistent throughout the manuscript.

19) Line 568. Khaki?
Corrected

20) Please prepare Table S2 according to the standards of the field and the Nature journals template.
Table S2 has now been formatted according to the standards of Nature journals and including the requests from the reviewer.

Some information should either be described in Methods or omitted. However, some critical information, for example, groupings of atoms, needs to be clarified. “Solvent” atoms are actually water molecules, while “other” atoms are apparently metals and nucleotides. It would be beneficial to have nucleotides and metals presented in separate categories.

In the revised version Table S2 has been modified according to the suggestions of the reviewer.

21) Table S2: Data collection and refinement statistics are not separated.
Changed as requested

22) Table S2. What value of C1/2 was used to determine the resolution cutoff? It is usually 0.5 (or 0.3 in some cases) for the highest resolution shell. The table lists 0.122 for the Nb8908 structure. Please see structural comments below. Please show the simulated annealing composite omit map for several key regions of the protein to demonstrate the quality of the map, which should match the expected resolution.

As recommended by the reviewers, this dataset was reprocessed to a CC1/2 of 0.39 (the statistics of the amended coordinates are in Table S2). In addition, Supplementary Fig. 3a-d shows the requested maps for Nb898, the HD domain, the SYNTH domain, and the complex interface, which constitute the key regions of the complex. In the zoomed panels, the side chains of residues involved in the complex interface are clearly visible in the electron density.

23) Line 705. Isothermal titration calorimetry
Corrected

24) Line 705-716. Please provide more details about ITC parameters and the experiments involving antibodies. How many times was each titration performed? There is no SD or SE in Supplementary Table 1.

We apologise for the omission. Each experiment was repeated 3 times, the errors (previously shown in the figures) are now shown in Table S1.

25) Line 718-738. Please provide a more detailed crystallization procedure here, including crystallization conditions. Were the structures obtained from the screening plates? If no, were screening plate conditions adjusted?
This information is now provided in the Methods section of the revised manuscript.

Crystals of Rel_{Ct}^{NTD} in complex with GDP and APCPP
"Crystals were obtained in the F8 condition of the LMB crystallization screen, 18 % w/v PEG 4000, 0.1 M Tris pH 9.0 and 0.3 M Sodium acetate trihydrate (Molecular Dimensions), at 20 °C using the sitting-drop vapour-diffusion technique with Swissci (MRC) 96-well 2-drop UVP sitting-drop plates. The 200 nL drops of 1:1 crystallization solution/protein sample, were deposited under humidity-controlled conditions using the Mosquito robotic system (TTP Labtech). The drops were equilibrated to 80 µL of the crystallization solution on the plate. For data collection, the crystals were directly harvested from the screening plate."

Crystals of Rel_{Ct}^{NTD} in complex with Nb898:
"Crystals were obtained in D9 condition of the LMB crystallization screen, 27 % w/v PEG 3350, 0.1 M Bis-Tris propane pH 7.0 and 0.2 M Li2SO4 (Molecular Dimensions) at 20 °C, using the sitting-

drop vapour-diffusion technique with Swissci (MRC) 96-well 2-drop UVP sitting-drop plates. The 200 nL drops of 1:1 crystallization solution/protein sample, were deposited under humidity-controlled conditions using the Mosquito robotic system (TTP Labtech). The drops were equilibrated to 80 µL of the crystallization solution on the plate. For data collection, the crystals were directly harvested from the screening plate."

26) Line 743. What is a composition of a “suitable” solution?

The word "suitable" has been removed, the sentence now reads:
"Prior to data collection, crystals the crystals were transferred to a cryoprotectant solution containing 38% of 2-Methyl-1,3-propanediol (MPD) and flash-cooled in liquid nitrogen."

27) How did the authors distinguish between Mg2+ and Mn2+ cations in the structures? Was it based on the anomalous map? The anomalous map for Mn1 is at the level of noise, and Mn5 does not have an anomalous map.

The assignment of metals was done based on prior knowledge of the binding sites. We appreciate the point made the reviewer, the Mn5 atom was mistakenly assigned, it should be Mg. This is now fixed in the revised structure.

28) There are numerous typos, especially in Methods.
Structures:
Nucleotide-bound structure
Corrected throughout.

29) The resolution cutoff of the structure needs further justification, as low CC1/2 of 0.411, along with poor completeness (53.5%) and subpar I/sI (1.1) in the highest resolution shell, suggests the overestimation of the resolution.

The resolution cutoff of the structure was estimated by STARANISO (https://staraniso.globalphasing.org) which was used to address the strong anisotropic diffraction of these crystals (this is the reason of the low spherical completeness). For the cutoff we used the standard CC1/2 0.3, well-established criteria for determining the resolution cut-off. Based on the quality of the maps we believe it has been correctly selected (moreover, these values are not unusual for datasets that are markedly anisotropic and are above accepted statistics).

30) Mg5, Mg6, and Mg7 in the nucleotide-bound structure are likely waters, as they contact a positively charged side chain and are located at the hydrogen bond distance, not a coordination distance of this metal. Please make sure that all metals are assigned correctly.

As suggested by the reviewer Mg5, Mg6, and Mg7 are now modelled as water molecules.

31) What is the huge unassigned map by Asn152/C and Mn4? Is this another nucleotide molecule? It is present in all molecules of the asymmetric unit.

There is indeed an unexplained density around Asn152. However, at the current resolution we cannot interpret it, and it is clearly not a nucleotide. Therefore we choose to leave it unassigned since further speculation will not contribute to the main findings and would cloud the message of the paper.

32) Some water molecules do not have 2Fo-Fc electron density map at 1.0 sigma level in both structures. What is the basis for modelling them?

The relative occupancy of every single water molecule is different and some might have a sigma level below 1.0. During the refinement water were initially placed automatically and then visually inspected validate their placement.

Antibody-bound structure
33) Rmerge of 0.619 indicates that the structure was not processed in the correct space group, or the resolution was significantly overestimated, as could be the case with very low CC1/2. I do not recall any published structure with such a high Rmerge at this resolution. I believe the maximum I saw was around 0.25. However, the electron density map is quite good. The authors mentioned the high anisotropy of the crystals (4.99 Å in b)* that could be a factor impacting the merging statistics. Was anisotropy correction performed? The authors should revisit their merging statistics, clarify this issue, and provide additional details on merging and choosing the resolution cutoff.

We remain confident in the assignment of the space group. As mentioned in the Methods section Staraniso was used correct for the strong anisotropic diffraction of these crystals as it is well-established in the field and it is likely as the reviewer mentions impacting the merging statistics.

34) The map is good, and there are no major issues with the structural model. In a few places, where the side chains were removed, I would leave some extra atoms, but this would not have an impact on the structure.

As pointed out, the resulting map is of high quality. This is now shown in the revised Supplementary Fig. As mentioned by the reviewer the choice of where to trim atoms in certain side chains is a personal one and have no impact on the structure and its analysis.

REPLY TO REVIEWERS

Reviewer #1 (Remarks to the Author)
Despite some minor flaws in the initial version, I had been enthusiastic about the manuscript in the first place (and obviously still am; keep up the work on the subject...). My remarks have been sufficiently addressed by the authors' improvements on the manuscript. That being said, I can now recommend publication.

We thank the reviewer for all the comments and suggestions that helped us improved the original version of the manuscript.

Reviewer #2 (Remarks to the Author)

The manuscript is greatly improved in its clarity. I only have a few additional minor suggestions:

Line 93: “stablished” should be changed to “established”.

Amended.

Lines 66-68: Missing Hogg et al 2004 (PMID: 15066282) which first showed reciprocal regulation of Syn and Hyd domains in long RSH

Introduced as requested.

Lines 114-117: Missing Pausch et al 2020 (PMID: 32937119) which is another structural study of off-ribosome Rel

Introduced as requested.

Lines 323-324: Missing Mechold et al 2002 (PMID: 32937119) which is the early work that shows CTD truncations in bifunctional long RSH increases Syn activity

Introduced as requested.

Reviewer #3 (Remarks to the Author)
The revised manuscript by Nerom et al. has been substantially improved. However, there are still a few overlooks that need to be addressed.
(1) All figures with ITC and Supplementary Table S1. What are Kds and errors (SD, SE) in each representative experiment in the figures? What are Kds and errors (SD, SE?) in each line of the table? How many times was each experiment repeated? This question has already been asked (point 24), but the authors failed to incorporate their answer into the manuscript.

Apologies for the overlook, in every case these are SD, each experiment was done in triplicates. This is now stated in the Methods section, Table S1 and Fig. legend.

(2) Fig. 2c,d. The statistics of the experiments are not defined in the figure legend. What are the values? Mean with SD?

Yes, mean and SD (amended throughout the manuscript).

(3) Point 30. Mg12/E is unlikely to be a Mg^{2+} cation as it contacts positively charged side chains of Arg and Lys. It is either an anion or a water molecule.

The final PDB has been amended as requested.

(4) Point 32. The authors' answer is confusing. I recommend incorporating the cutoff criteria for automatic picking of water molecules, as some waters are unlikely to be picked up by, for example, Phenix's default parameters. However, Buster apparently picked them up. For example, HOH29/G has literally no density. Although water occupancy may vary, the density must be well above the noise. Several water molecules have a density at sigma level 0.5, basically the noise level.

In the revised coordinates, all water molecules are now above a sigma level of 1.3.

(5) Point 22. SFig. 4. In the legend, please specify which density map is presented in panels a-c.

Modified as requested

In panels g, h, and I, are these maps simulated annealing composite omit maps as in the response to point 22? "Unbiased omit maps" is not a sufficient description.

Yes, these are simulated annealing composite omit maps. The complete wording has been included now in the legend.

(6) Fig. 4c, d. Please specify the type of map contour level in the legend.

This information was already present in the figure, but earlier in the sentence. We modified it in the revised version to include the wording "simulated annealing composite omit maps…".

(7) Fig. 4 legend. ITC is panel e, not d.

The legend is correct; the only issue is that each panel containing an ITC titration is cited after the corresponding individual titration is mentioned.

(8) Fig. 5a. Please indicate by a red dashed line box the region with increased deuterium uptake (K47-R48, etc.).

Modified as requested.

(9) Fig. 5b-d. A graph for "a control" peptide, without much uptake, such as residue 130-137, must be shown here.

Three different control peptides for regions : 128-135; 136-143; 233-243, have been included in the figure as requested by the reviewer.

(10) Fig. 5a. The R.F.U. scale for the top two images is not shown here.

Amended.

(11) I think Figs. 1rev, 2rev, and Fig. 4rev could be incorporated into the supplementary Information section.

As suggested, all three figures are now included in SFig. 3.

(12) Point 18. Fig. 6. Most colors in panel a look darker than in panels b and c, especially red, pink, and yellow. Please label domains. It is difficult to go back to Fig. 1a for color-coding. To avoid confusion, perhaps antibodies should not be shaded here, as they are not shown in the cartoon. Initially, I thought that they were presented in the structures, and many readers would assume the same.

We prefer to keep this layout. It follows the color-coding used throughout the manuscript starting from Fig. 1.

(13) Point 20. Table. S3. The need for atom-grouping is to show B-factors for each category, especially for metals and nucleotides, as they are not highly occupied. If occupancy is overestimated, the atoms will be “hot”. This is still not done here despite the rebuttal letter saying that this issue was addressed. There are still “Other atoms” in B-factor section. A footnote should specify which ligands are included, as nucleotides are not the only ligands in the structures.

Supplementary Table S3 has been modified as requested and now lists the B-factors of ligands separately (as metals and nucleotides).
